# Endothelial deletion of the neddylation E2 enzyme UBE2M inhibits tumor growth by promoting nonproductive angiogenesis

**DOI:** 10.1172/JCI201769

**Published:** 2026-07-21

**Authors:** Xinyi Jiang, Jie Zhang, Li Zhou, Zonglin Li, Ningcong Sun, Xian Xu, Jisong Zhang, Yizhou Huang, Xue Zhang, Enguo Chen, Hongqiang Cheng, Yuehai Ke

**Affiliations:** 1 Department of Pathology and Pathophysiology and Department of Respiratory Medicine, Sir Run Run Shaw Hospital,; 2 Department of Urology, Sir Run Run Shaw Hospital,; 3 Department of Pathology and Pathophysiology and Department of Cardiology of Sir Run Run Shaw Hospital,; 4 Department of Respiratory and Critical Care Medicine, Regional Medical Center for National Institute of Respiratory Diseases, Sir Run Run Shaw Hospital, and; 5 Department of Gynecology, Women’s Hospital, Zhejiang University School of Medicine, Hangzhou, China.; 6 Zhejiang University–University of Edinburgh Institute (ZJE) Cross Lung Research Alliance, International Joint Center for Biomedical Sciences, Zhejiang University, Haining, China.; 7 State Key Laboratory of Transvascular Implantation Devices, Hangzhou, China.

**Keywords:** Cell biology, Vascular biology, Angiogenesis, Cancer, Endothelial cells

## Abstract

Neddylation is highly activated in many human cancers and may serve as a therapeutic target for clinical treatment. However, the role of neddylation in tumor angiogenesis remains unclear. Here, we demonstrate that the neddylation E2 enzyme UBE2M was upregulated in tip cells and was essential for tumor vascular sprouting. We showed that UBE2M-mediated neddylation of STAT1 enhanced its phosphorylation and promoted the transcription of DLL4. This elevated DLL4 expression in tip cells activated Notch signaling in adjacent stalk cells, thereby maintaining the tip-stalk cell balance and ensuring organized vascular patterning. Consequently, endothelial cell–specific deletion of UBE2M reduced DLL4 expression, leading to excessive but nonproductive sprouting due to uncontrolled tip cell formation and lack of stalk cell support, which ultimately suppressed tumor growth. Importantly, targeting endothelial neddylation potently sensitized various tumors to anti-VEGF therapy. Together, our findings unveil UBE2M as a key regulator of angiogenic signaling and identify it as a promising antiangiogenic target in cancer.

## Abstract 2

Endothelial UBE2M deletion reduces DLL4 and disrupts tumor vascular patterning, inducing non-productive angiogenesis that suppresses tumor growth.

## Introduction

The progression and metastasis of solid tumors critically rely on the establishment of a neovascular network, which provides oxygen, nutrients, and metabolic waste exchange and serves as routes for metastatic spread (1). Consequently, targeted inhibition of tumor angiogenesis represents a highly promising therapeutic strategy in oncology (2, 3). Angiogenesis arises from existing vasculature through sprouting, a process dynamically balanced by pro- and antiangiogenic factors (4). VEGF is a pivotal proangiogenic factor, and drugs targeting the VEGF pathway — such as bevacizumab and small-molecule tyrosine kinase inhibitors — constitute a mainstay of clinical antiangiogenic therapy (5, 6). However, despite their widespread use, these agents often elicit limited therapeutic efficacy due to acquired resistance and primary nonresponse across many tumor types (7–10). Developing more effective antiangiogenic strategies therefore remains a critical and challenging goal in cancer research.

The core mechanism of sprouting angiogenesis lies in the precise regulation of tip and stalk cell specification (11). Tip cells, characterized by high motility and abundant filopodia, guide sprout extension, while stalk cells form the vascular lumen through proliferation and interconnection (12). Research indicates that tip and stalk cells dynamically compete and interconvert in response to VEGF gradients, ensuring efficient and organized sprouting — a process coordinated by VEGF and Notch signaling (13). VEGF upregulates DLL4 expression in tip cells, which activates Notch in adjacent endothelial cells, suppresses VEGFR2 expression, and promotes stalk cell fate (14). Notably, inhibition of DLL4/Notch signaling in tumors leads to nonproductive angiogenesis, characterized by excessive but poorly perfused vessels, ultimately resulting in suppressed tumor growth (15–17).

Posttranslational modifications represent one of the key mechanisms regulating signal transduction and cellular behaviors. Given that neddylation is highly activated in numerous cancers, it has become an increasing focus of research interest (18–24). As a dynamic and reversible ubiquitin-like modification, the neddylation system consists of the NEDD8-activating enzyme E1 (a heterodimer of NAE1/APPBP1 and UBA3), NEDD8-conjugating enzyme E2 (UBE2M and UBE2F), multiple E3 ligases, and NEDD8-specific proteases (including sentrin-specific protease 8 [SENP8] and JAB1/CSN5) (25, 26). During neddylation, the C-terminal glycine residue of NEDD8 is covalently attached via an isopeptide bond to the ε-amino group of a lysine residue on the target protein. While its canonical role involves the activation of Cullin-RING ubiquitin ligases, a growing number of non-Cullin substrates have been identified, implicating neddylation in an expanding range of biological functions (27–34). Emerging evidence has elucidated the critical regulatory roles of neddylation in endothelial homeostasis. Previous studies have demonstrated that neddylation mediates activation of the Cullin3-CRL3 ubiquitin ligase complex to modulate VE-cadherin stability and maintain endothelial barrier function (35). Neddylation of HDAC6 also contributes to vascular tone and atherogenesis regulation (36). However, the functions and mechanisms of neddylation in tumor angiogenesis remain largely elusive and require further investigation.

In this study, we discovered that endothelial neddylation promotes the phosphorylation of transcription factor STAT1 through direct modification, thereby modulating the expression of DLL4. This regulatory axis controlled the specification of tip and stalk cell phenotypes during tumor angiogenic sprouting. Furthermore, inhibition of endothelial neddylation sensitized diverse tumors to anti-VEGF therapy. The combination of the two therapies increased pericyte coverage alongside elevated vascular density, which may account for the enhanced tumor inhibition. This finding provides a strategic perspective for clinical antiangiogenic treatment.

## Results

### UBE2M is highly expressed in tumor endothelial cells.

**Figure 1:**
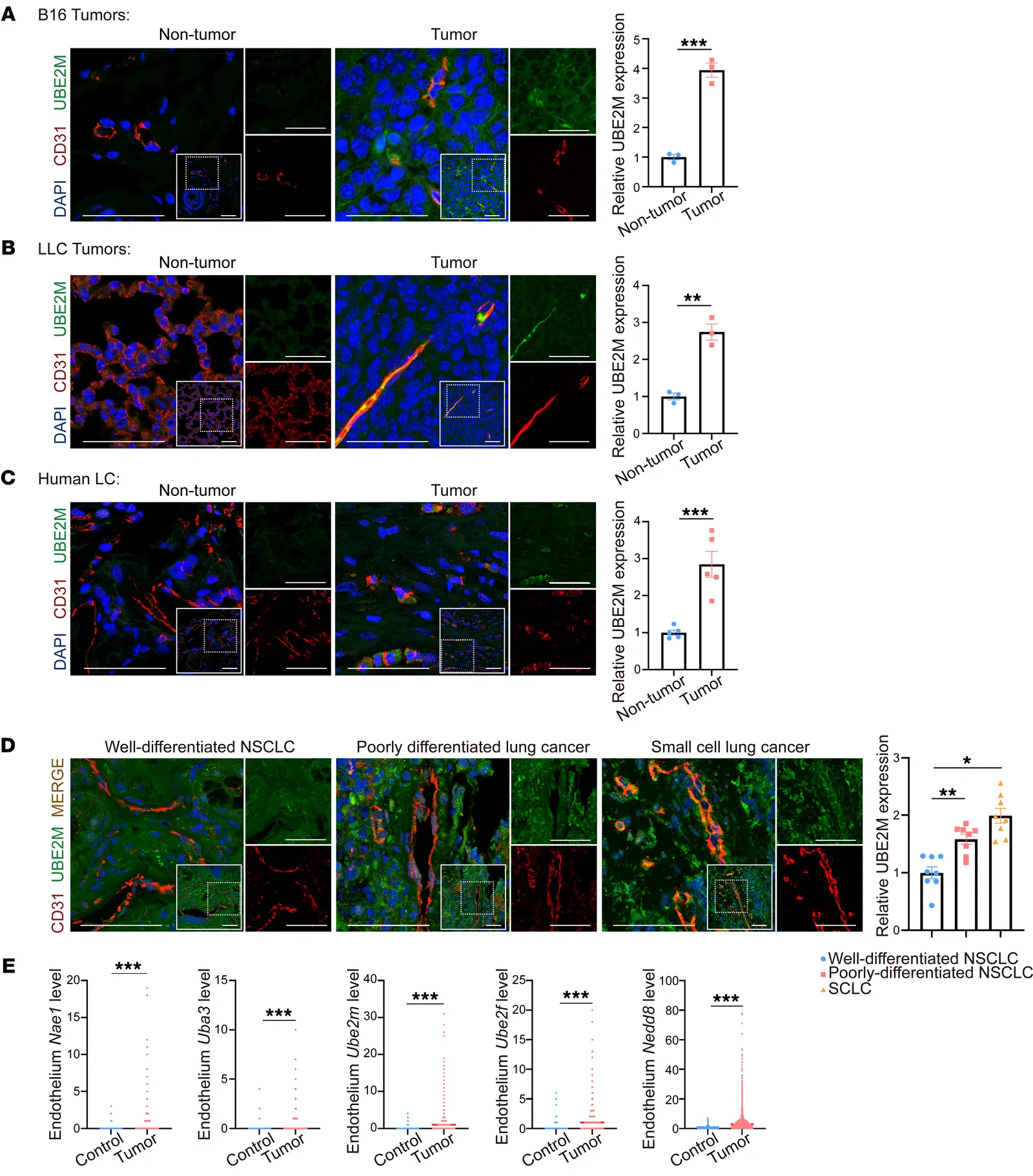
UBE2M is highly expressed in tumor endothelial cells. (**A** and **B**) Representative images of UBE2M in CD31^+^ vessels from B16 melanoma (**A**) and LLC tumor (**B**) and matched normal tissues (*n* = 3). Scale bar: 50 μm. (**C**) Representative immunofluorescence staining images of UBE2M expression in tumor endothelial cells of lung cancer (*n* = 5). Scale bar: 50 μm. (**D**) Representative immunofluorescence staining images of UBE2M expression in tumor endothelial cells of well-differentiated NSCLC, poorly differentiated lung carcinoma, and small cell lung carcinoma (*n* = 8). Scale bar: 50 μm. (**E**) Expression of neddylation-related genes (*Nae1*, *Uba3*, *Ube2m*, *Ube2f*, *Nedd8*) in mouse normal and tumor endothelial cells was extracted from the GEO dataset (GSE118904). Data are shown as mean ± SEM. *P < 0.05; **P < 0.01; ***P < 0.001. Two-tailed Student’s *t* test (**A**–**C** and **E**); 1-way ANOVA followed by Tukey’s post hoc test (**D**).

Comparative analysis revealed elevated UBE2M expression in platelet endothelial cell adhesion molecule-1–labeled (CD31-labeled) tumor-associated endothelial cells versus nontumor endothelium in murine B16 melanoma and Lewis lung carcinoma (LLC) models (Figure 1, A and B). We also found that UBE2M expression was upregulated in the endothelial cells of human lung cancers relative to nontumor lung tissues (Figure 1C). Next, clinical lung cancer tissues across malignancy grades were collected for immunofluorescence staining. UBE2M was upregulated in the tumor vasculature of hypervascular lung cancer subtypes (Figure 1D). Furthermore, analysis of a murine dataset (GSE118904, RNA-seq of tumor vs. nontumor endothelial cells) also revealed higher expression of multiple neddylation pathway genes, including UBE2M, in tumor-associated endothelial cells (Figure 1E). These results collectively demonstrate markedly elevated UBE2M expression in tumor endothelial cells.

### Endothelial cell–specific UBE2M deletion restricts tumor growth.

**Figure 2:**
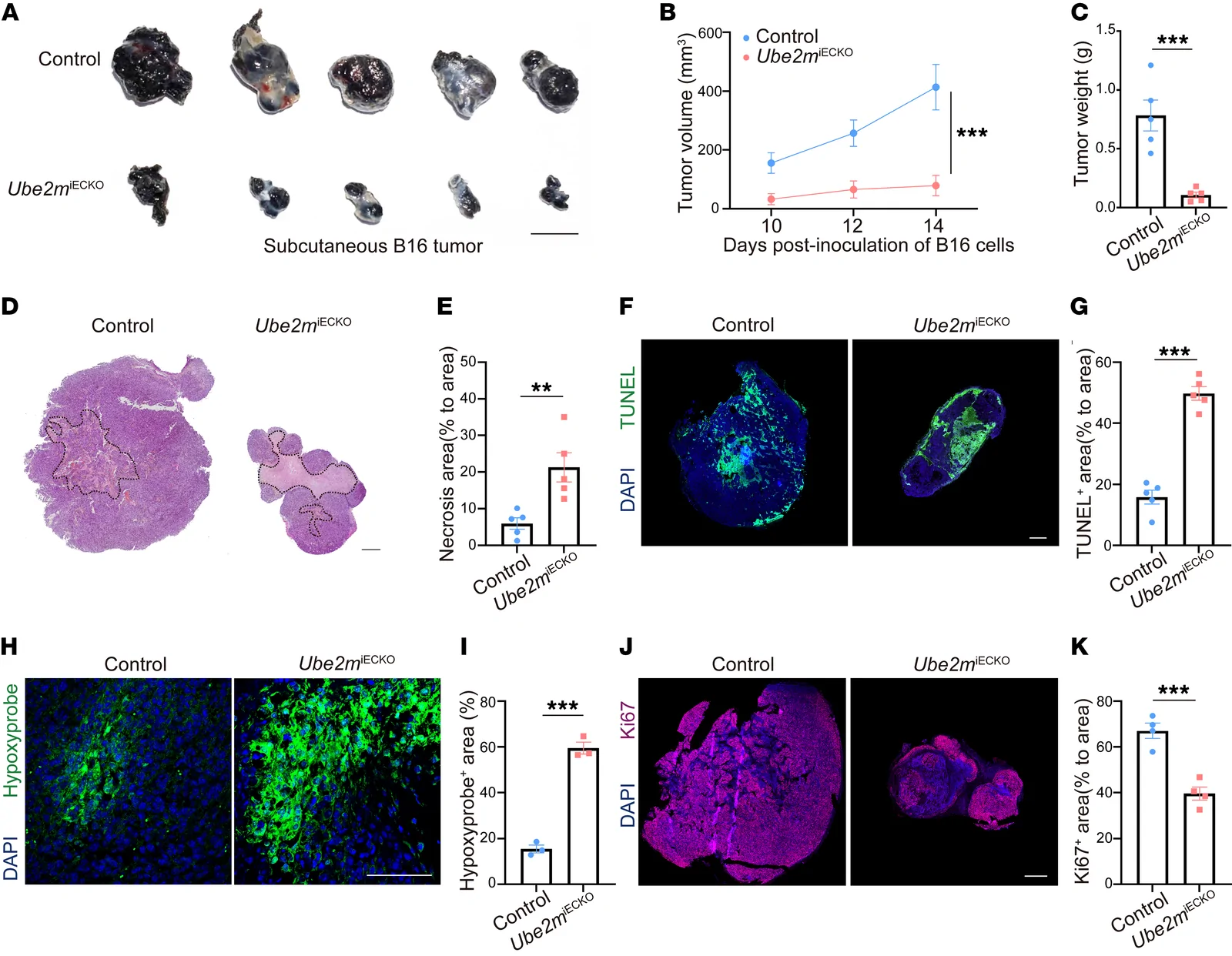
Endothelial UBE2M deletion suppresses tumor growth. (**A**) Images of subcutaneous B16 melanoma tumors from *Ube2m*^fl/fl^ and *Ube2m*^iECKO^ mice (*n* = 5). Scale bar: 10 mm. (**B** and **C**) The volumes (**B**) and weights (**C**) of B16 tumors in *Ube2m*^fl/fl^ and *Ube2m*^iECKO^ mice (*n* = 5). (**D**–**G**) Representative images of B16 tumor necrosis (**D** and **E**) and apoptosis (**F** and **G**). H&E staining was conducted and the necrosis area was labeled with dotted lines (*n* = 5). Apoptosis was measured by TUNEL staining (*n* = 5). Scale bar: 1 mm. (**H** and **I**) Representative images of hypoxyprobe-1–labeled areas in B16 tumors (*n* = 3). Scale bar: 100 μm. (**J** and **K**) Representative images of Ki67^+^ areas in B16 tumors (*n* = 4). Scale bar: 1 mm. Data are shown as mean ± SEM. **P < 0.01; ***P < 0.001. Two-tailed Student’s *t* test (**C** and **D**–**K**); 2-way ANOVA followed by Tukey’s post hoc test (**B**).

We first demonstrated that UBE2M deficiency in the endothelium does not alter the vascular structure of multiple organs (heart, liver, spleen, lung, and kidney) (Supplemental Figure 1, A–C; supplemental material available online with this article; https://doi.org/10.1172/JCI201769DS1). To elucidate the role of UBE2M in tumor-associated vasculature, conditional endothelial UBE2M-knockout mice (*Ube2m*^iECKO^) were generated. Efficient endothelial UBE2M depletion was achieved through the tamoxifen-inducible Cre-loxP system (Supplemental Figure 2, A and B). One week after the final tamoxifen injection (tamoxifen was given for 5 days by i.p. injection), 20 mg/kg/d), B16 tumor cells were subcutaneously inoculated into *Ube2m*^iECKO^ and *Ube2m*^fl/fl^ mice to assess tumor growth. The results demonstrated that endothelial cell–specific UBE2M deficiency significantly suppressed subcutaneous melanoma growth (Figure 2, A–C). Consistent with melanoma findings, UBE2M depletion in tumor vasculature attenuated orthotopic E0771 breast cancer growth (Supplemental Figure 2, C–E). In addition, we assessed the effect of UBE2M deficiency on subcutaneous LLC tumor growth. Unexpectedly, LLC tumor progression was unaffected by endothelial UBE2M deletion (Supplemental Figure 2, F–H). Notably, endothelial UBE2M ablation markedly increased intratumoral necrosis, apoptosis, and hypoxic areas, while suppressing tumor cell proliferation (Figure 2, D–K). This tumor-suppressive phenotype, particularly the enhanced necrosis and apoptosis, was also seen in orthotopic E0771 tumors (Supplemental Figure 2, I–L) but was absent in the subcutaneous LLC model (Supplemental Figure 2, M–P).

### Endothelial UBE2M deletion causes aberrant excessive vascularization.

**Figure 3:**
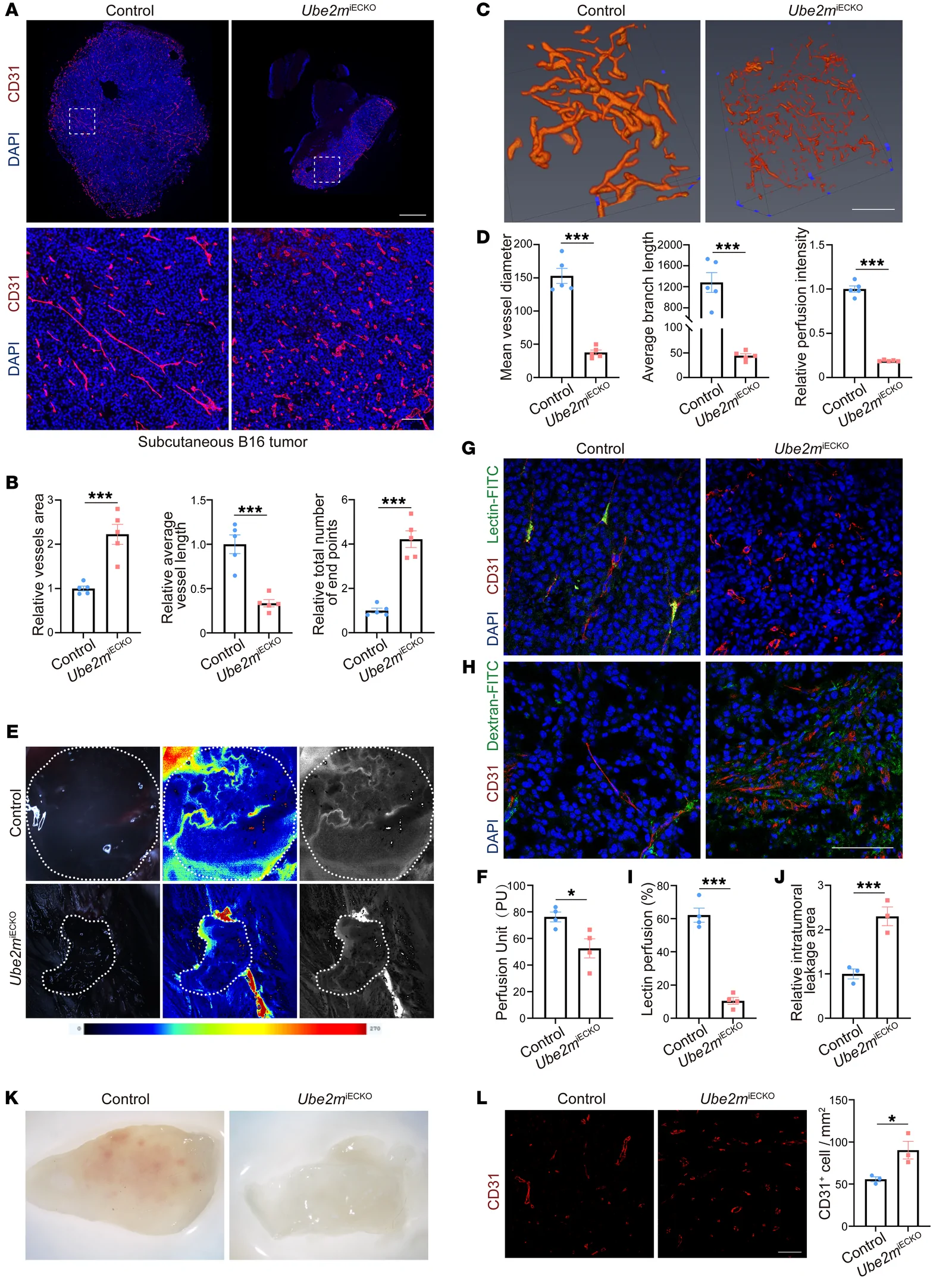
Endothelial deletion of UBE2M promotes nonproductive angiogenesis. (**A** and **B**) Immunofluorescence images of CD31 in B16 tumors from *Ube2m*^fl/fl^ and *Ube2m*^iECKO^ mice (*n* = 5), showing an overview (top) and a higher-magnification view (bottom) of the boxed region. Scale bars: 1 mm (top), 100 μm (bottom). Images were analyzed by ImageJ software. (**C** and **D**) Representative 3D rendered images of tumor vasculature in B16 tumors from *Ube2m*^fl/fl^ and *Ube2m*^iECKO^ mice via CT imaging. Scale bar: 1 mm. (**E** and **F**) Laser speckle contrast imaging of blood flow in B16 tumors in *Ube2m*^fl/fl^ and *Ube2m*^iECKO^ mice (*n* = 4). (**G**–**J**) Representative images for vessels perfused with lectin (**G** and **I**) and dextran (**H** and **J**) in B16 tumors from *Ube2m*^fl/fl^ and *Ube2m*^iECKO^ mice (*n* = 4 per group for lectin; *n* = 3 per group for dextran). Images were analyzed by ImageJ software. Scale bar: 100 μm. (**K** and **L**) Images of Matrigel plugs from *Ube2m*^fl/fl^ and *Ube2m*^iECKO^ mice and immunofluorescence images of CD31 in plugs from *Ube2m*^fl/fl^ and *Ube2m*^iECKO^ (*n* = 3). Scale bar: 50 μm. Images were analyzed by ImageJ software. Data are shown as mean ± SEM. *P < 0.05; ***P < 0.001. *P* values were generated by using the 2-tailed Student’s *t* test.

To define how endothelial UBE2M depletion restricts tumor progression, we evaluated the tumor microvasculature in *Ube2m*^iECKO^ and control mice. Immunofluorescence analysis revealed significantly increased CD31^+^ microvascular area and density but reduced average vessel length in the tumors of *Ube2m*^iECKO^ mice (Figure 3, A and B). Micro-CT angiography further revealed that endothelial UBE2M-deficient tumors exhibited increased vascular density and branching complexity, characterized by marked vascular fragmentation and narrowed vessel diameters (Figure 3, C and D). Moreover, endothelial UBE2M deletion consistently increased vascular density while reducing average vessel length in both orthotopic breast cancer and subcutaneous LLC models (Supplemental Figure 3, A–D). Furthermore, tumors from endothelial UBE2M-deficient mice exhibited reduced pericyte coverage (labeled by α-SMA and NG2) and impaired perivascular basement membrane integrity, as indicated by collagen IV staining, collectively indicating vascular abnormality (Supplemental Figure 4, A–C).

### Inhibition of endothelial UBE2M promotes nonproductive angiogenesis.

The observed tumor suppression despite increased vascular density prompted us to investigate the functionality of the tumor vasculature. Laser speckle contrast imaging revealed impaired tumor perfusion in *Ube2m*^iECKO^ mice compared with that in control mice (Figure 3, E and F). Lectin perfusion assays revealed impaired vascular perfusion in tumors of endothelial UBE2M-deficient mice (Figure 3, G and I), along with increased dextran leakage, indicating hyperpermeability (Figure 3, H and J). Similarly, endothelial UBE2M deficiency increased vascular density yet reduced lectin perfusion and exacerbated vessel leakage in both orthotopic E0771 mammary tumors (Supplemental Figure 3, E–H) and subcutaneous LLC tumors (Supplemental Figure 3, I–L). The lack of significant growth inhibition by endothelial UBE2M deletion in subcutaneous LLC tumors might be attributed to the adaptation to this vascular phenotype, as existing literature suggests that LLC tumors are inherently less sensitive to certain antiangiogenic therapies (37, 38). To isolate endothelial cell–specific effects and exclude tumor cell contributions, we employed in vivo Matrigel plug angiogenesis assays. At day 7 after implantation, Matrigel plugs in control mice showed visible red coloration, while *Ube2m*^iECKO^ plugs were markedly pale, indicating impaired blood perfusion (Figure 3K). However, immunofluorescence analysis revealed more CD31^+^ endothelial cells in plugs of *Ube2m*^iECKO^ mice compared with controls (Figure 3L), recapitulating the phenotype observed in tumors. Despite the presence of nonproductive angiogenesis induced by endothelial UBE2M deficiency, characterized by hypoperfusion and hyperpermeability, our analysis of the tumor immune microenvironment revealed no significant effect on F4/80^+^ macrophage recruitment or cytotoxic T cell activity (GZMB^+^ area) (Supplemental Figure 4, D and E). These observations indicated that the observed vascular dysfunction does not necessarily lead to broad immunosuppression in this context. These results indicated that endothelial UBE2M deletion induced nonproductive angiogenesis, ultimately impairing tumor progression.

### UBE2M-mediated neddylation in tip cells maintains angiogenic sprouting homeostasis.

**Figure 4:**
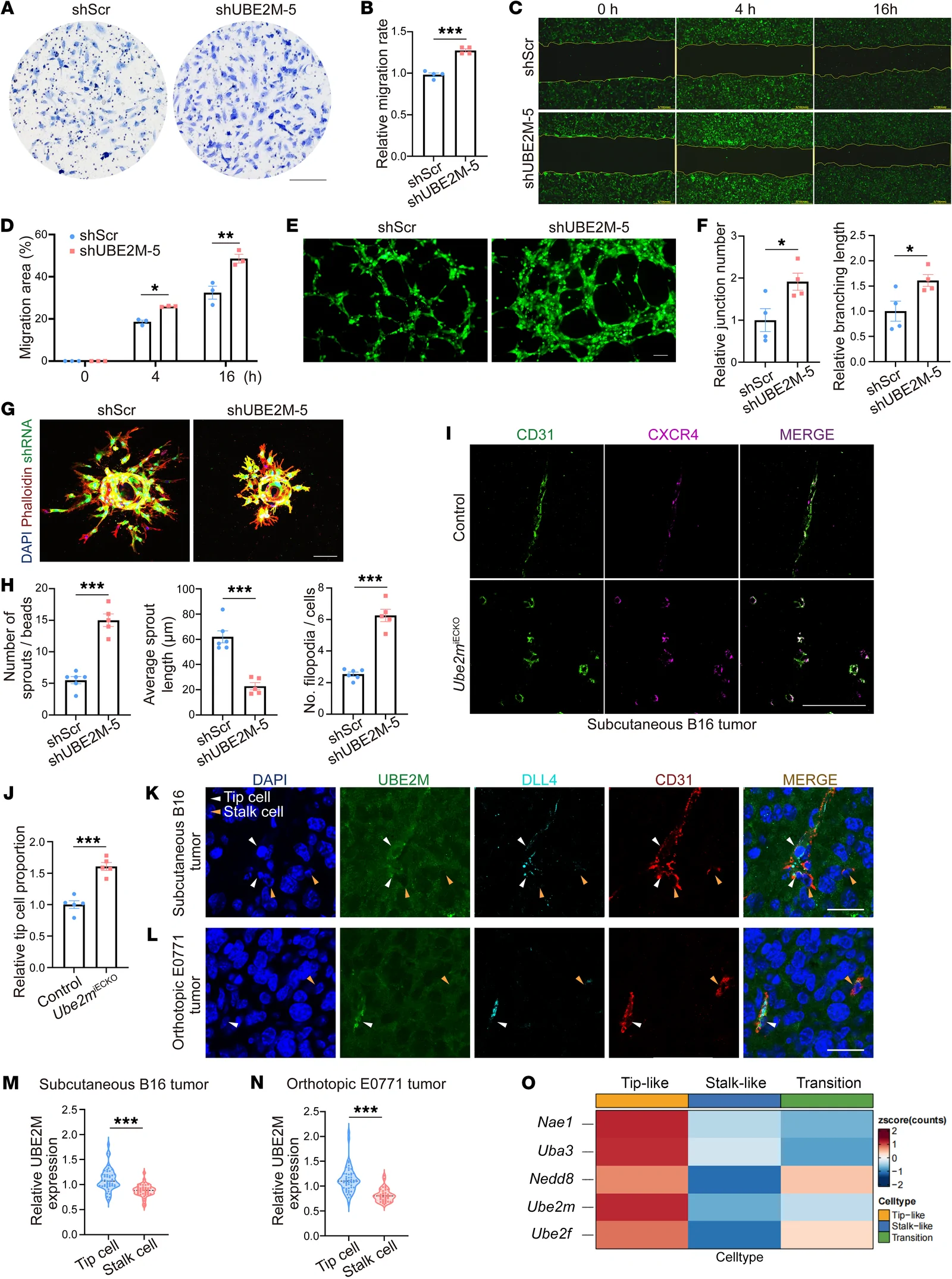
UBE2M deficiency in endothelial cells leads to excessive tip cell induction. (**A**–**D**) Cell migration was measured by Transwell migration (**A** and **B**; *n* = 4, Scale bar: 200 μm) and in vitro wound healing assay of HUVECs (C and D; n = 3, scale bar: 500 μm). (**E**–**G**) Angiogenic function measured by the tube formation (**E** and **F**; *n* = 4, Scale bar: 100 μm) and sprouting assay (**G** and **H**, *n* = 6 for shScr, *n* = 5 for shUBE2M-5, Scale bar: 100 μm). Images were measured using ImageJ software. (**I** and **J**) Representative images showing CXCR4^+^ tip cells in CD31^+^ vessels in B16 melanoma tumor tissues (*n* = 5). Scale bar: 50 μm. The proportion of CXCR4^+^ cells among CD31^+^ endothelial cells was quantified. (**K**–**N**) Representative images showing UBE2M expression (green) in tip cells (DLL4^+^, CD31^+^) and stalk cells (DLL4^–^, CD31^+^) in subcutaneous B16 (**K** and **M**) and orthotopic E0771 (**L** and **N**) tumor tissues (*n* = 3 for B16 tumor; *n* = 3 for E0771 tumor). Scale bar: 20 μm. UBE2M expression in tip cells and stalk cells was analyzed by ImageJ software. (**O**) Comparative expression analysis of neddylation pathway–related genes across the identified EC subtypes. The heatmap depicts *z*-score scaled expression levels, highlighting subtype-specific neddylation activity. Data source: GSE159013. Quantitative data are shown as mean ± SEM. *P < 0.05; **P < 0.01; ***P < 0.001. Two-tailed Student’s *t* test (**B**, **F**, **H**, **J**, **M**, and **N**); 2-way ANOVA followed by Tukey’s post hoc test (**D**).

Given that tumors with endothelial UBE2M deficiency exhibited markedly increased vascular density, reduced average vessel length, and exacerbated hypoxia, we first examined the proliferation and apoptosis status of tumor endothelial cells. Ki67 and CD31 costaining, as well as TUNEL and CD31 costaining, showed that UBE2M deficiency did not affect endothelial cell proliferation or apoptosis (Supplemental Figure 5, A and B). To further investigate the cellular mechanism of UBE2M in angiogenesis, we transfected HUVECs with shRNA to knock down UBE2M and examined endothelial cell angiogenic capabilities. CCK-8 (cell counting kit-8) assay demonstrated that UBE2M knockdown/knockout (with efficiencies verified in Supplemental Figure 5, C and D, for HUVECs and Supplemental Figure 2, A and B, for mouse lung endothelial cells) did not affect endothelial cell proliferation (Supplemental Figure 5, E and F). Wound healing and Transwell assays revealed that UBE2M knockdown enhanced endothelial cell migration, while cells with UBE2M overexpression exhibited loss of migration ability (Figure 4, A–D, and Supplemental Figure 6, A and B). Similarly, both UBE2M knockdown and neddylation inhibitor MLN4924 treatment enhanced tube formation in HUVECs, as evidenced by increased branching length and junction number (Figure 4, E and F, and Supplemental Figure 6, E and F). Although MLN4924 was previously shown to inhibit angiogenesis and tube formation (39–41), we found that low-dose MLN4924 effectively inhibited neddylation and enhanced tube formation to the same extent as UBE2M knockdown (Supplemental Figure 6, C–F). These findings suggest that the inhibition effects on tube formation observed at higher concentrations may result from nonspecific drug toxicity rather than targeted neddylation inhibition. Next, we assessed the sprouting ability of UBE2M-knockdown HUVECs using a sprouting assay. UBE2M depletion enhanced endothelial sprouting while simultaneously reducing average sprout length and increasing the number of filopodia per cell (Figure 4, G and H). Additionally, shUBE2M ECs formed more phalloidin^+^ filopodia in a scratch-wound model (Supplemental Figure 6, G and H). These results indicated that UBE2M knockdown in endothelial cells causes excessive tip cell formation coupled with insufficient stalk cell proliferation to support proper luminal extension. This model of excessive tip cell formation was directly confirmed in vivo, where histological analyses revealed an increased proportion of tip cells, identified by the established markers CXCR4 (42) and CD34 (43), in both tumors and plugs in endothelial cell–specific UBE2M deletion mice (Figure 4, I and J, and Supplemental Figure 6I). Based on these findings, we propose that the lateral inhibition exerted by tip cells is dependent on their own neddylation modification. Further immunofluorescence analyses demonstrated elevated UBE2M expression in tip cells across multiple tumor types (Figure 4, K–N). Additionally, analysis of a single-cell transcriptomic dataset from tumor endothelium (GSE159013), clustered using established markers (44) (Supplemental Figure 7, A–C), consistently showed notably higher expression of key neddylation components (NAE1, UBA3, UBE2M, UBE2F, and NEDD8) in tip cells (Figure 4O). Collectively, these data establish UBE2M-mediated neddylation as a critical regulator of tumor vascular sprouting. It maintains the balance between tip and stalk cells, and its loss disrupts this balance, promoting excessive tip cell formation and ultimately leading to nonproductive angiogenesis that suppresses tumor growth.

### UBE2M regulates the expression of DLL4 in the endothelium.

**Figure 5:**
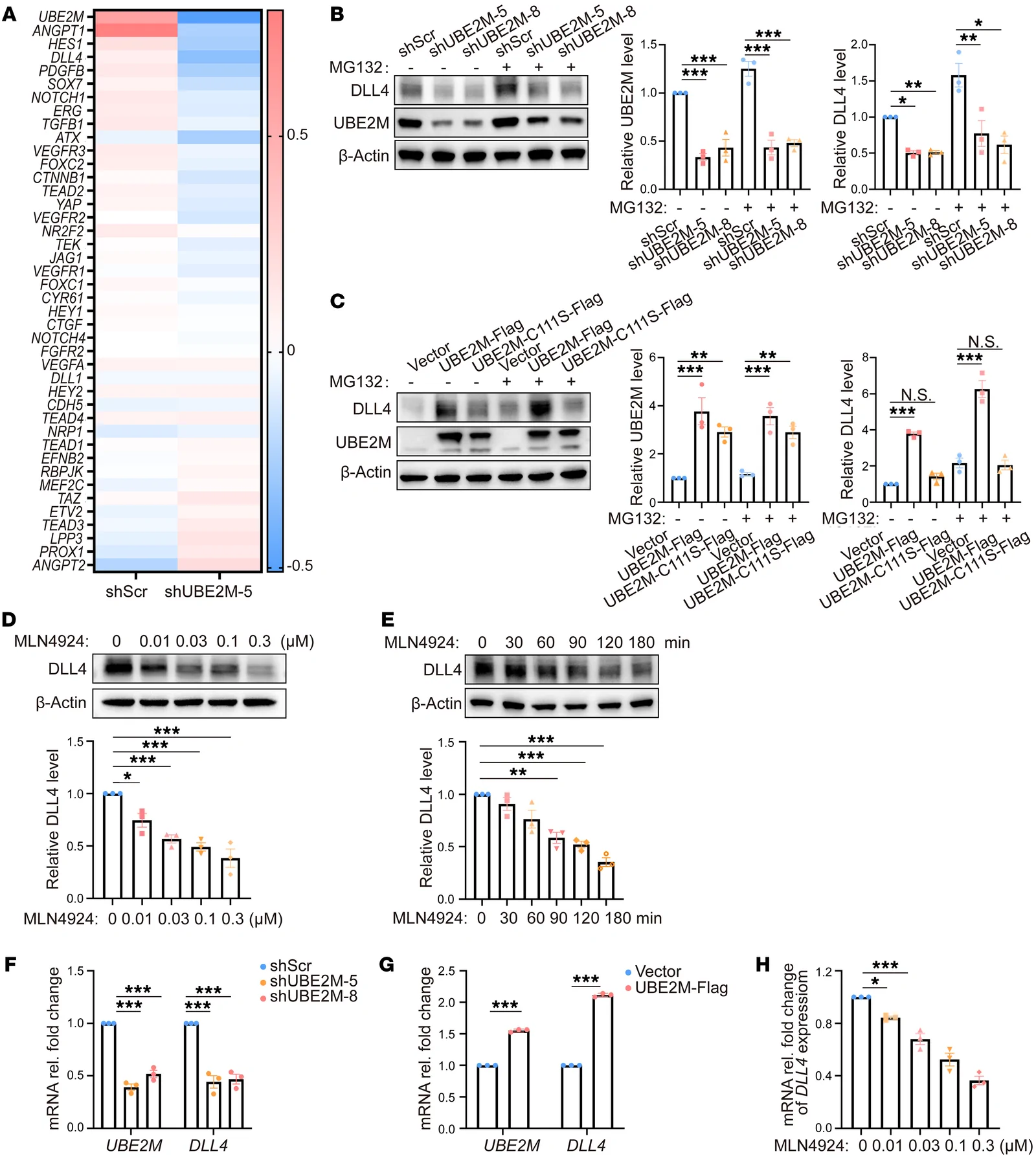
UBE2M regulates DLL4 expression. (**A**) Heatmap showing qPCR results of angiogenesis-related genes in UBE2M knockdown (shUBE2M) and control (shScr) HUVECs. (**B**) Western blot for DLL4 expression in UBE2M-knockdown and control HUVECs (*n* = 3). β-Actin was used as a loading control. (**C**) Western blot for DLL4 in overexpression of UBE2M or its enzymatic dead mutant (UBE2M-C111S) and control HUVECs (*n* = 3). β-Actin was used as a loading control. (**D** and **E**) Western blot analysis of DLL4 expression in HUVECs treated with MLN4924 at concentration and time gradients (*n* = 3). (**F**–**H**) *DLL4* expression was analyzed by qPCR in HUVECs subjected to UBE2M knockdown (**F**), UBE2M overexpression (**G**), or MLN4924 treatment (**H**). Quantitative data are shown as mean ± SEM. *P < 0.05; **P < 0.01; ***P < 0.001; nonsignificant, *P* > 0.05. One-way ANOVA followed by Tukey’s post hoc test (**B**–**E**, **H**); 2-way ANOVA followed by Tukey’s post hoc test (**F**, **G**).

**Figure 6:**
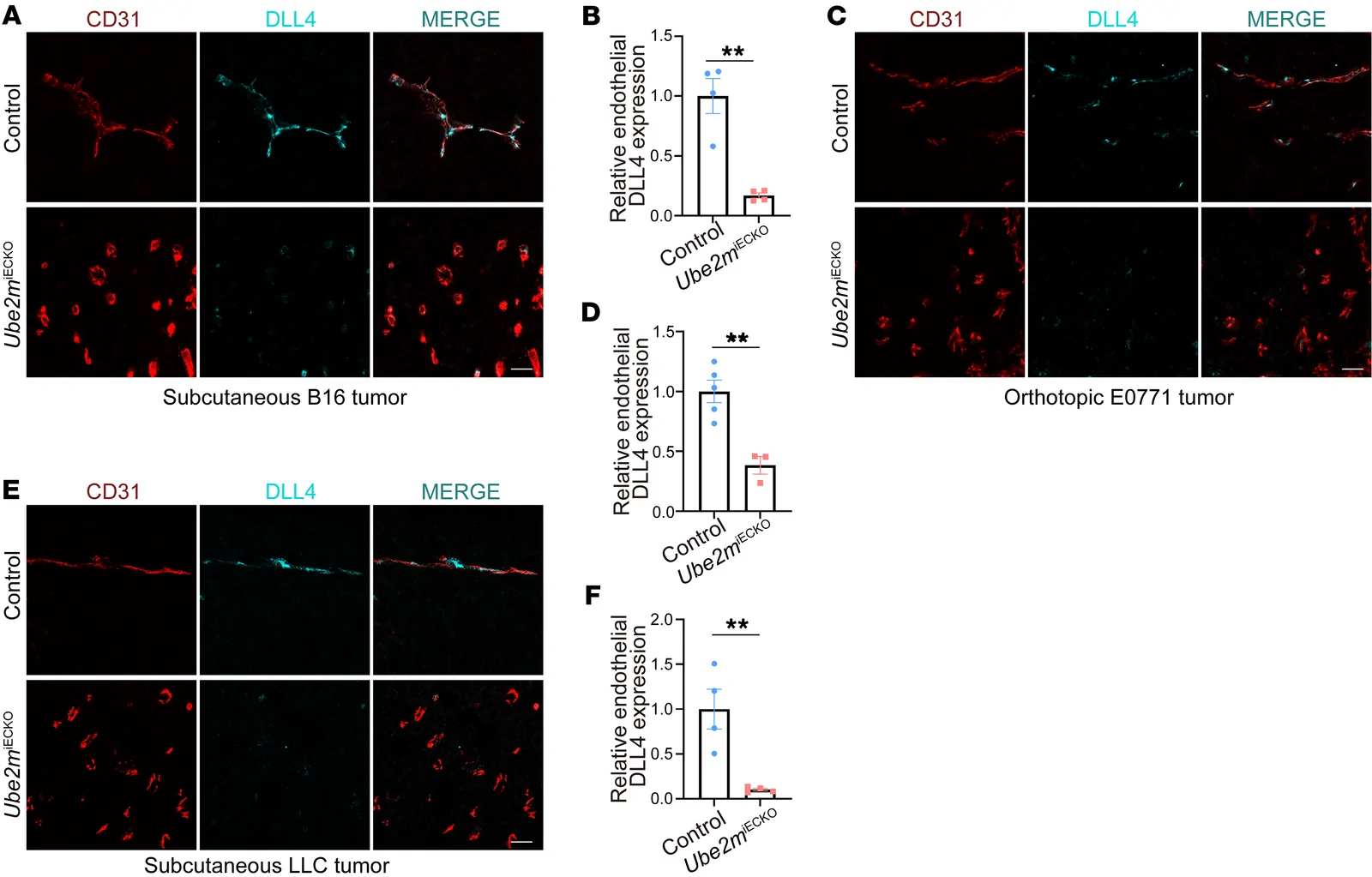
UBE2M deficiency reduces DLL4 expression in tumor endothelial cells. (**A** and **B**) Representative images showing DLL4 expression in CD31^+^ vessels of subcutaneous B16 tumors from *Ube2m*^fl/fl^ and *Ube2m*^iECKO^ mice (*n* = 4). Scale bar: 20 μm. (**C** and **D**) Representative images showing DLL4 expression in CD31^+^ vessels of orthotopic E0771 tumors from *Ube2m*^fl/fl^ and *Ube2m*^iECKO^ mice (*n* = 5 for *Ube2m*^fl/fl^ and *n* = 3 for *Ube2m*^iECKO^ mice). Scale bar: 20 μm. (**E** and **F**) Representative images showing DLL4 expression in CD31^+^ vessels of subcutaneous LLC tumors from *Ube2m*^fl/fl^ and *Ube2m*^iECKO^ mice (*n* = 4). Scale bar: 20 μm. Endothelial DLL4 was analyzed by ImageJ software. Quantitative data are shown as mean ± SEM. *P < 0.05; **P < 0.01; ***P < 0.001. Two-tailed Student’s *t* test (**B**, **D**, and **F**).

To investigate the molecular mechanisms by which UBE2M regulates tumor angiogenesis and endothelial cells, we analyzed the expression of angiogenesis-related genes in UBE2M-knockdown and control HUVECs. UBE2M knockdown in ECs inhibited the expression of HES1 and DLL4 (Figure 5A). Since HES1 is a downstream effector of DLL4/Notch signaling and based on our in vivo and in vitro phenotypic data, we focused on the DLL4 gene. Western blot analysis revealed that UBE2M knockdown reduced DLL4 expression in HUVECs, whereas overexpression of UBE2M, but not its enzymatic dead mutant (UBE2M-C111S), markedly increased DLL4 levels (Figure 5, B and C). Moreover, MG132-mediated proteasome inhibition had no significant effect on UBE2M-dependent DLL4 expression changes, suggesting that UBE2M regulates DLL4 at the transcriptional level. Similarly, MLN4924 treatment of HUVECs reduced DLL4 expression in a both time- and dose-dependent manner (Figure 5, D and E). As expected, both UBE2M knockdown and MLN4924 treatment reduced *DLL4* mRNA levels in HUVECs, whereas WT UBE2M overexpression markedly increased DLL4 transcription (Figure 5, F–H). To validate these results in vivo, immunofluorescence staining was performed, revealing reduced DLL4 expression in endothelial cells of *Ube2m*^iECKO^ mice across different tumor models, including subcutaneous B16 melanoma (Figure 6, A and B), orthotopic E0771 breast cancer (Figure 6, C and D), and subcutaneous LLC tumor (Figure 6, E and F) when compared with control mice. Thus, UBE2M modulates DLL4 expression.

### DLL4 reexpression in tumor endothelial cells restored vasculature and tumor growth in Ube2m^iECKO^ mice.

**Figure 7:**
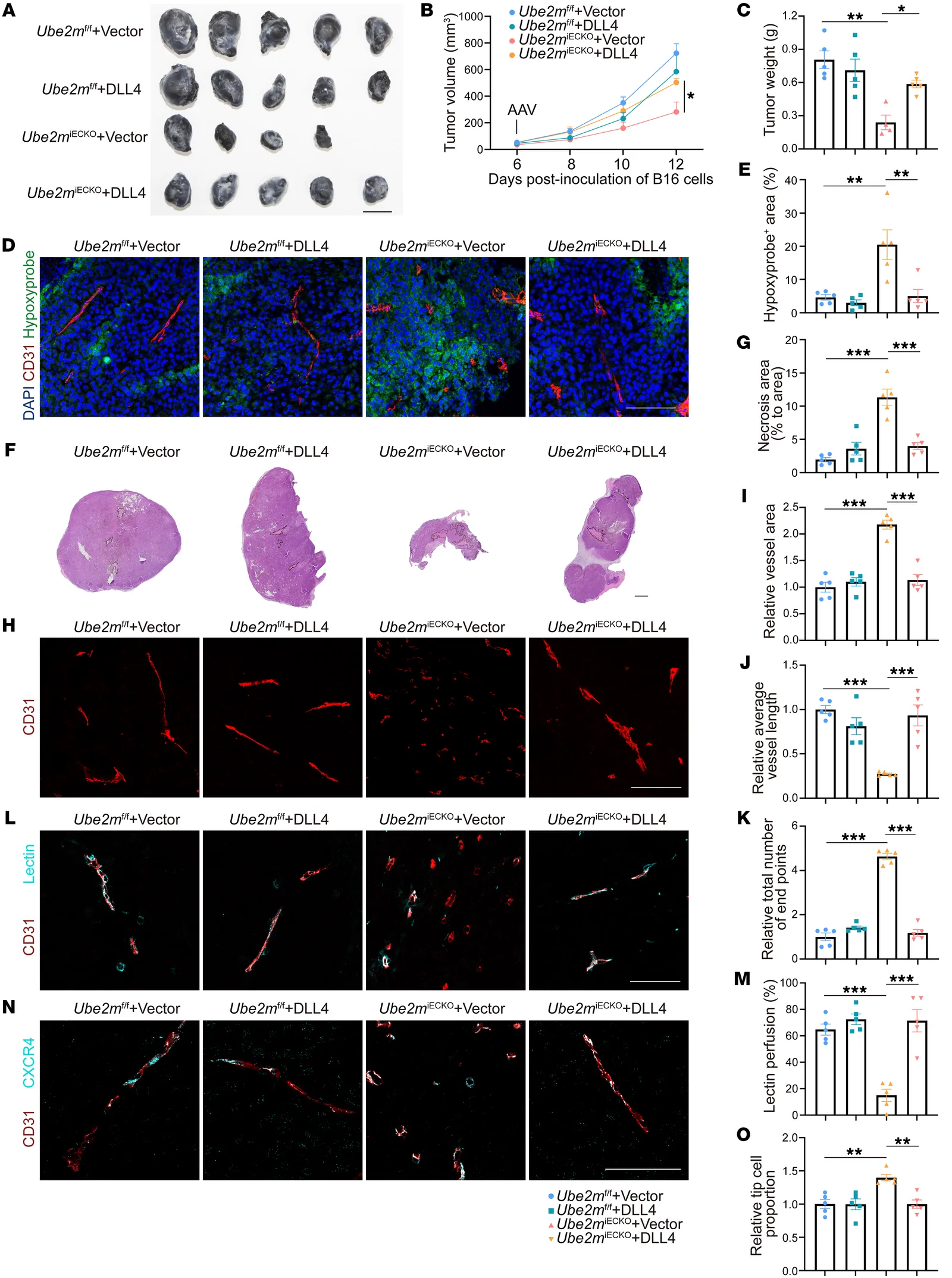
DLL4 reexpression in tumor endothelial cells restored tumor growth and vasculature in *Ube2m*^iECKO^ mice. (**A**) Images of B16 melanoma tumors from *Ube2m*^fl/fl^ and *Ube2m*^iECKO^ mice treated with AAV-DLL4. *n* = 5 per group (*n* = 4 for *Ube2m*^iECKO^) Scale bar: 10 mm. (**B** and **C**) Tumor volumes (**B**) and tumor weights (**C**) of B16 tumors from *Ube2m*^fl/fl^ and *Ube2m*^iECKO^ mice treated with AAV-DLL4. Arrows indicated the time points for AAV and tamoxifen injection. Tumor weights were measured 12 days after cancer cell injection. (**D** and **E**) Representative images of CD31 staining and hypoxyprobe-1–labeled areas in B16 tumors. Images were analyzed by ImageJ software (*n* = 5). Scale bar: 100 μm. (**F** and **G**) Representative images of B16 tumor necrosis. Necrosis areas were labeled by dotted lines. Images were analyzed by ImageJ software (*n* = 5). Scale bar: 1 mm. (**H**–**K**) Immunofluorescence images of CD31 (**H**) in B16 tumors. Vessel area (**I**), average vessel length (**J**), and total number of end points (**K**) were measured by ImageJ software (*n* = 5). Scale bar: 100 μm. (**L** and **M**) Immunofluorescence images of CD31 and lectin in B16 tumors. Images were measured by ImageJ software (*n* = 5). Scale bar: 50 μm. (**N** and **O**) Immunofluorescence images of CD31 and CXCR4 in B16 tumors. The proportion of CXCR4^+^ cells among CD31^+^ endothelial cells was quantified (*n* = 5). Scale bar: 50 μm. Data are shown as mean ± SEM. *P < 0.05; **P < 0.01; ***P < 0.001. Two-way ANOVA with Tukey’s post hoc test (**B**) or 1-way ANOVA with multiple comparisons (**C**, **E**, **G**, **I**–**K**, **M**, and **O**).

In vitro, both DAPT treatment and UBE2M knockdown similarly disrupted endothelial sprouting (Supplemental Figure 8A). Like UBE2M knockdown, DLL4 knockdown also enhanced phalloidin^+^ filopodia formation in HUVECs in a scratch-wound model (Supplemental Figure 6, G and H). DLL4 reexpression in UBE2M-deficient HUVECs rescued tube formation capacity to control levels (Supplemental Figure 8, B and C), with the knockdown of UBE2M and reexpression of DLL4 verified by Western blotting (Supplemental Figure 8D). To rescue angiogenesis defects caused by UBE2M deletion, a Cre-responsive AAV vector was constructed for endothelial cell–specific DLL4 expression. CD31 and ZsGreen1 costaining demonstrated effective and specific AAV expression in tumor endothelial cells of *Ube2m*^iECKO^ mice, and DLL4 coimmunofluorescence staining further confirmed DLL4 protein expression in these cells of the *Ube2m*^iECKO^-DLL4 group (Supplemental Figure 8, E and F). DLL4 reexpression in tumor vascular endothelial cells restored tumor growth (Figure 7, A–C), along with a reduction in intratumoral hypoxia (Figure 7, D and E) and necrosis area (Figure 7, F and G) in *Ube2m*^iECKO^ mice. Notably, DLL4 expression in *Ube2m*^iECKO^ mice reduced tumor vessel area while increasing average vessel length (Figure 7, H–K), ultimately restoring tumor vascular perfusion (Figure 7, L and M). Furthermore, endothelial DLL4 reexpression reduced the proportion of tip cells in the tumor vasculature of *Ube2m*^iECKO^ mice (Figure 7, N and O). In the main, these successful rescue experiments demonstrated that DLL4 is the critical downstream effector of UBE2M in tumor angiogenesis.

### Neddylation promotes the activity of the DLL4 transcription factor STAT1.

**Figure 8:**
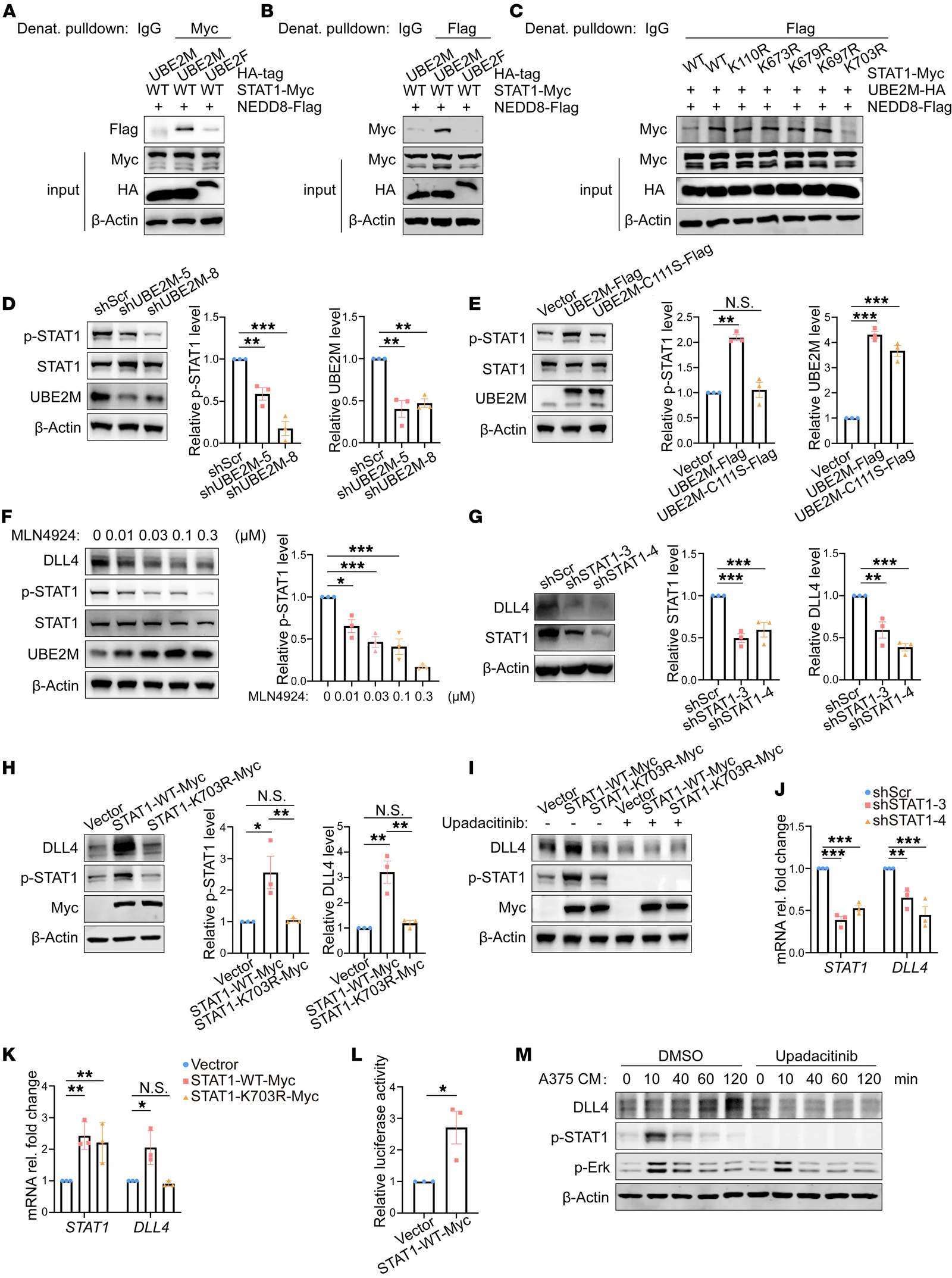
Neddylation promotes the activity of the DLL4 transcription factor STAT1. (**A**) Western blot analysis of Myc (STAT1) pulldown in HEK293T cells overexpressing STAT1, NEDD8, and UBE2M/UBE2F. Denat., denaturation. (**B**) Western blot analysis of Flag (NEDD8) pulldown in HEK293T cells overexpressing STAT1, NEDD8, and UBE2M/UBE2F. (**C**) Immunoblot analysis of proteins immunoprecipitated with an anti-Flag antibody from HEK293 cells transfected with the indicated STAT1 mutants. (**D**) Western blot in UBE2M-knockdown and control HUVECs (*n* = 3). β-Actin was used as a loading control. (**E**) Western blot analysis of lysates from HUVECs overexpressing UBE2M, its enzyme-dead mutant (UBE2M-C111S), as well as control cells (*n* = 3). β-Actin was used as a loading control. (**F**) Western blot analysis of HUVECs treated with a concentration gradient of MLN4924 (*n* = 3). β-Actin was used as a loading control. (**G**) Western blot analysis of DLL4 and STAT1 in STAT1-knockdown and control HUVECs (*n* = 3). β-Actin served as a loading control. (**H**) Western blot analysis of DLL4 and p-STAT1 in HUVECs overexpressing STAT1 and STAT1-K703R mutant as well as control cells (*n* = 3). β-Actin served as a loading control. (**I**) Western blot of p-STAT1 and DLL4 in HUVECs overexpressing STAT1-WT or STAT1-K703R after upadacitinib treatment. (**J**) qPCR for *DLL4* in STAT1-knockdown HUVECs (*n* = 3). (**K**) qPCR for *DLL4* in HUVECs overexpressing STAT1-WT or the STAT1-K703R mutant. (**L**) Luciferase reporter assay in HEK293 cells with STAT1 overexpression. The data were normalized to the Renilla luciferase activity. (**M**) Western blot analysis of DLL4 and p-STAT1 levels in HUVECs stimulated with A375 CM in the presence of upadacitinib. Data are shown as mean ± SEM for 3 independent experiments. *P < 0.05; **P < 0.01; ***P < 0.001; nonsignificant, *P* > 0.05. Two-tailed Student’s *t* test (**L**); 2-way ANOVA followed by Tukey’s post hoc test (**J** and **K**); 1-way ANOVA followed by Tukey’s post hoc test (**D**–**H**).

To identify NEDD8-modified proteins that potentially regulate endothelial DLL4 expression, we performed mass spectrometry (MS) to screen for NEDD8-conjugated proteins in HUVECs. The results indicated that NEDD8 interacts with Cullins, NAE1, UBE2M, and β-catenin in endothelial cells, all of which were previously reported to be enzymes or substrates of protein neddylation, confirming the reliability of our MS results (Supplemental Figure 9, A and B). Furthermore, potential transcription factors of DLL4 were identified using 3 transcription factor databases (Supplemental Figure 9C). Among these factors, STAT1 and SMAD2 were identified as neddylation substrates by MS. Subsequent denaturing immunoprecipitation (IP) assay further verified that STAT1 was neddylated by UBE2M (Figure 8, A and B). Potential neddylation sites in STAT1 were predicted using Neddypreddy (45), identifying 5 lysine residues (K110, K673, K679, K697, and K703) as putative neddylation sites (Supplemental Figure 9D). Further denaturing IP assays demonstrated that the K703R mutation virtually abolished neddylation, suggesting that Lys703 is the predominant neddylation site on STAT1 (Figure 8C).

To elucidate the functional effect of neddylation on STAT1, we conducted both knockdown and overexpression of UBE2M in HUVECs. The results demonstrated that UBE2M knockdown suppressed STAT1 phosphorylation at Tyr701 (Figure 8D), while overexpression of WT UBE2M but not UBE2M-C111S enhanced the phosphorylation (Figure 8E). Additionally, MLN4924 treatment in a concentration-dependent manner reduced STAT1 phosphorylation levels (Figure 8F). This regulatory relationship was further confirmed in vivo, as *Ube2m*^iECKO^ mice exhibited markedly reduced p-STAT1 levels in tumor-associated endothelial cells (Supplemental Figure 9, E and F).

Next, we further validated the transcriptional regulation of DLL4 by STAT1. In HUVECs, STAT1 knockdown reduced, while its overexpression increased, both DLL4 protein (Figure 8, G and H) and mRNA (Figure 8, J and K) levels. Critically, this upregulation depended on STAT1 neddylation, as the neddylation-deficient mutant STAT1-K703R was ineffective. Furthermore, the JAK1/2 inhibitor upadacitinib abolished the STAT1-induced DLL4 expression (Figure 8I). Subsequently, luciferase reporter assays demonstrated that STAT1 overexpression in HEK293 cells enhanced DLL4 transcriptional activity compared with that in control groups (Figure 8L). Additionally, we discovered that tumor-conditioned medium stimulation promoted STAT1 phosphorylation and upregulated DLL4 expression in HUVECs. Treatment with upadacitinib simultaneously suppressed both STAT1 phosphorylation and DLL4 elevation (Figure 8M). Our results demonstrate that UBE2M mediates STAT1 neddylation at Lys703, which in turn promotes STAT1 phosphorylation at Tyr701, ultimately enhancing DLL4 expression in endothelial cells.

### Targeting endothelial UBE2M inhibits tumor growth and improves the sensitivity of tumors to antiangiogenic treatment.

**Figure 9:**
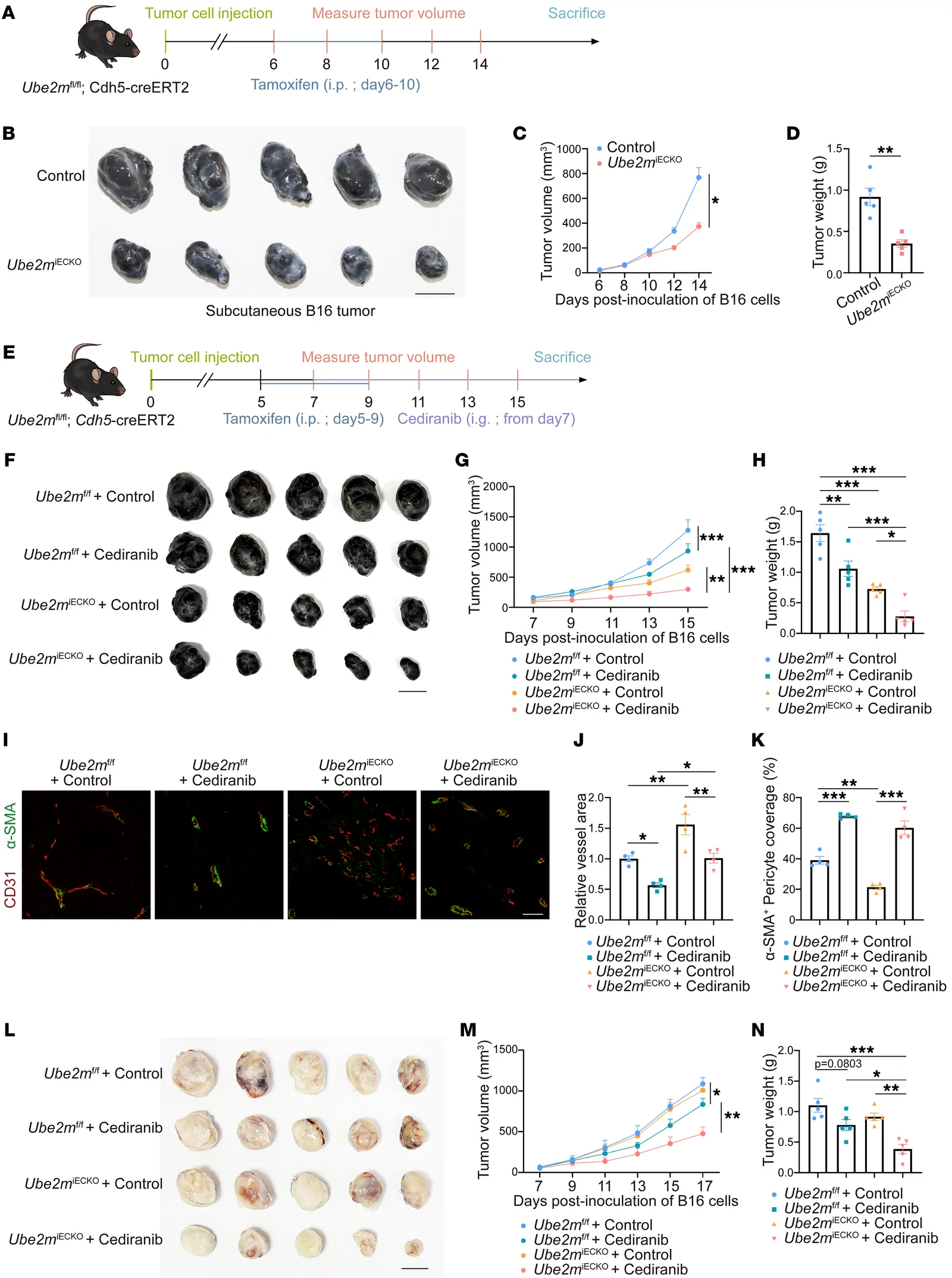
Targeting endothelial UBE2M inhibits tumor growth and sensitizes to anti-VEGF treatment. (**A**) Model of inducible UBE2M knockout after tumor establishment. Schematic timeline: Tumors were allowed to grow in *Ube2m*^fl/fl^ and *Ube2m*^iECKO^ mice until they reached 5 mm in diameter, at which point tamoxifen was administered to delete UBE2M in the knockout group. (**B**) Images of subcutaneous B16 tumors from *Ube2m*^fl/fl^ and *Ube2m*^iECKO^ mice (*n* = 5). Scale bar: 10 mm. (**C** and **D**) The volumes (**C**) and weights (**D**) of B16 tumors from *Ube2m*^fl/fl^ and *Ube2m*^iECKO^ mice. (**E**) Schematic of combined inducible UBE2M knockout and cediranib treatment after tumor establishment. (**F**) Images of subcutaneous B16 tumors from *Ube2m*^fl/fl^ and *Ube2m*^iECKO^ mice treated with cediranib (*n* = 5). Scale bar: 10 mm. (**G** and **H**) The volumes (**G**) and weights (**H**) of B16 tumors from *Ube2m*^fl/fl^ and *Ube2m*^iECKO^ mice treated with cediranib. (**I**–**K**) Immunofluorescence images showing CD31 and α-SMA in B16 tumors from *Ube2m*^fl/fl^ and *Ube2m*^iECKO^ mice (*n* = 4) Scale bar: 50μm. (**L**) Images of subcutaneous LLC tumors from *Ube2m*^fl/fl^ and *Ube2m*^iECKO^ mice treated with cediranib (*n* = 5). Scale bar: 10 mm. (**M** and **N**) The volumes (**M**) and weights (**N**) of LLC tumors from *Ube2m*^fl/fl^ and *Ube2m*^iECKO^ mice treated with cediranib. *P < 0.05; **P < 0.01; ***P < 0.001. Two-tailed Student’s *t* test (**D**); 2-way ANOVA followed by Tukey’s post hoc test (**C**, **G**, and **M**); 1-way ANOVA followed by Tukey’s post hoc test (**H**, **J**, **K**, and **N**).

To better reflect clinical therapeutic scenarios, we initially examined whether UBE2M deficiency in endothelial cells retains its antitumor efficiency against established tumors. i.p. tamoxifen injection was performed to induce endothelial cell–specific UBE2M knockout when subcutaneous tumors reached 5 mm in diameter (Figure 9A). Consistent with the results presented in Figure 2, A–C (UBE2M knockout before tumor cell injection), endothelial cell–specific UBE2M deficiency also exhibited a pronounced antitumor effect against established tumors (Figure 9, B–D).

In clinical antiangiogenic cancer therapy, targeting VEGF signaling represents a well-established approach. To further investigate the combined antitumor effect of endothelial UBE2M deletion and anti-VEGF therapy, we injected tamoxifen to knockout endothelial UBE2M when tumor reached 5 mm, followed by daily treatment with the VEGFR2 inhibitor (cediranib) (Figure 9E). In the melanoma model, endothelial cell–specific UBE2M deficiency produced an additive inhibitory effect on tumor growth when combined with anti-VEGF therapy, as evidenced by a significantly smaller tumor volume and weight in the combination group compared with either monotherapy (Figure 9, F–H). Immunofluorescence staining revealed that, compared with anti-VEGF monotherapy, the combination therapy led to tumors with higher vascular density, and compared with UBE2M deletion alone, it resulted in increased α-SMA^+^ pericyte coverage (Figure 9, I–K). Next, in the subcutaneous LLC model, consistent with the results shown in Supplemental Figure 2F (UBE2M knockout before tumor cell injection), endothelial cell–specific UBE2M deficiency did not significantly inhibit established LLC tumor growth, while anti-VEGF treatment alone showed moderate inhibitory effects. Surprisingly, anti-VEGF treatment in endothelial UBE2M-deficient mice demonstrated enhanced inhibition of LLC tumor growth compared with anti-VEGF treatment alone (Figure 9, L–N).

We next examined the effect of combined treatment with the neddylation inhibitor (MLN4924) and the VEGFR2 inhibitor (cediranib) on tumor growth. In the subcutaneous B16 melanoma, treatment with MLN4924 or cediranib alone moderately inhibited tumor growth, whereas the combination of both drugs resulted in further enhanced suppression of tumor progression (Supplemental Figure 10, A–C). In the orthotopic E0771 breast cancer, combined treatment with MLN4924 and cediranib also reduced tumor volume compared with either monotherapy (Supplemental Figure 10, D and E). A tendency toward reduced tumor weight was noted in the combination group, although this difference did not reach statistical significance when compared individually to the monotherapy groups, likely due to considerable intragroup variation in tumor weight measurements (Supplemental Figure 10F). Collectively, these results demonstrate that combined treatment with MLN4924 and cediranib produces enhanced antitumor efficacy, indicating that targeting endothelial neddylation is an effective strategy to suppress tumor growth and sensitize it to anti-VEGF therapy. Combined with the observation that UBE2M deficiency does not affect normal organ vasculature, these results support targeting endothelial neddylation as a potential therapeutic strategy for antiangiogenic cancer therapies.

## Discussion

Numerous studies have implicated the upregulation of neddylation in tumorigenesis across various cancers, highlighting its potential as a therapeutic target. This upregulation has been extensively documented in various solid tumors, including lung (46, 47), breast (30, 48, 49), and colorectal cancers (50), and is frequently associated with poor patient prognosis (25). Such dysregulation leads to the excessive activation of Cullin-RING ligases (CRLs), leading to the degradation of tumor suppressors that drives cancer progression, as exemplified by neddylation-mediated stabilization of HER2 in breast cancer (30) and degradation of MEN1 in pancreatic neuroendocrine tumors (51). Notably, the regulatory role of neddylation extends beyond cancer cells and is deeply involved in remodeling the tumor microenvironment — for example, by modulating macrophage function to mediate immunosuppression (33) or by influencing metabolic reprogramming to facilitate environmental adaptation (52). In this study, we identified an upregulation of neddylation pathway components in tumor-associated endothelial cells, where this pathway critically maintains vascular sprouting homeostasis to support the formation of functional vasculature.

Tumor vascular patterning relies on VEGF-driven competition and dynamic tip-stalk interconversion; this process is balanced by VEGF, which promotes the tip cell state, and Notch signaling, which mediates lateral inhibition to suppress it in neighboring cells (13, 53–56). In hypoxic regions of tumors, the HIF-1α signaling pathway promotes VEGF expression to activate tip cells and upregulates the expression of the stress-inducible gene *UBE2M* in endothelial cells — a finding consistent with earlier reports in cancer cells (57). Moreover, VEGF itself has been shown to promote UBE2M expression in HUVECs in a time- and concentration-dependent manner (41). These findings suggest the elevated expression of neddylation-related genes in hypoxic and VEGF-activated tip cells, as confirmed by our immunofluorescence staining and single-cell transcriptomic analyses. This state is linked to interendothelial Notch signaling, forming an adaptive feedback mechanism. As cell identities change, the neddylation profile is accordingly reprogrammed to precisely meet the functional demands, thereby maintaining controlled vascular sprouting. This reveals neddylation as a previously overlooked mechanism for ensuring the ordered sprouting of vasculature.

**Figure 10:**
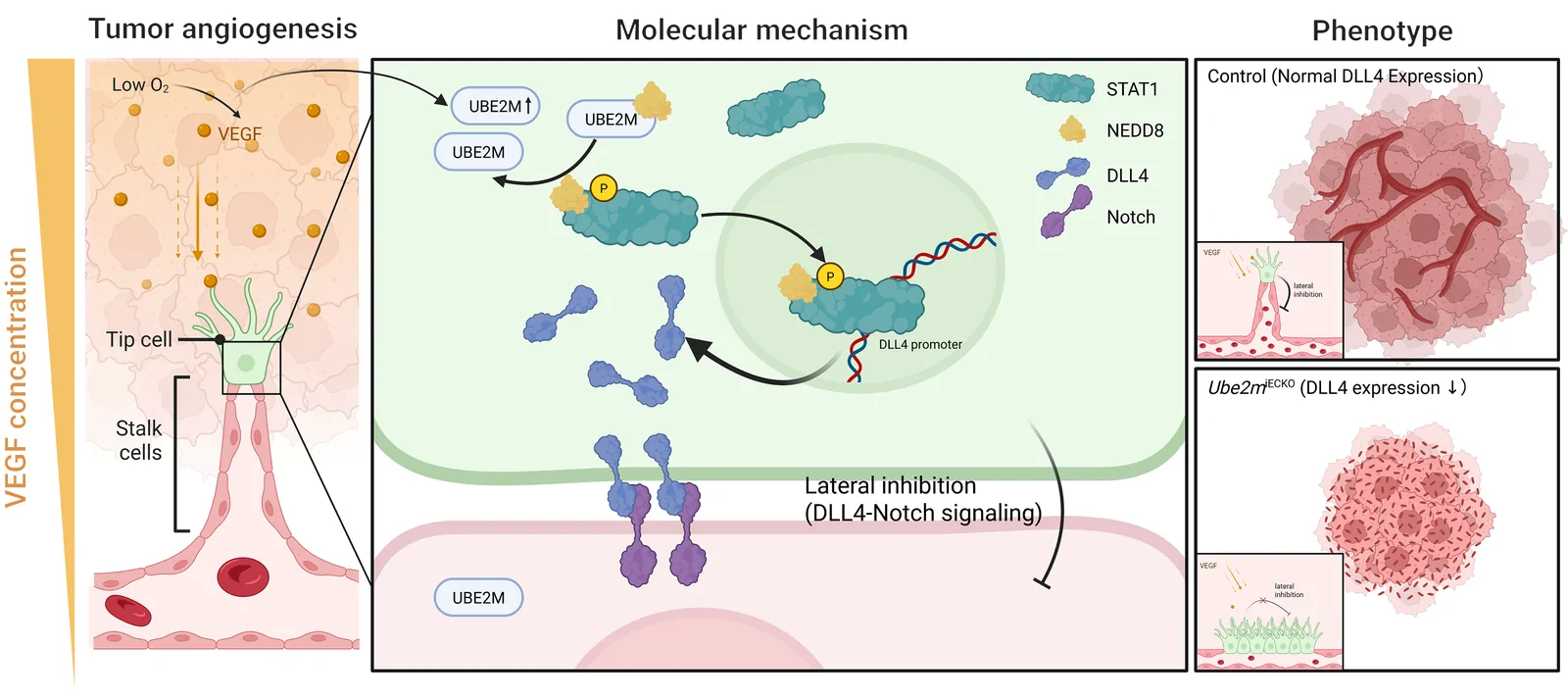
Mechanistic model for UBE2M-mediated tumor angiogenesis. Proposed model of UBE2M-mediated regulation of tip and stalk cell fate and nonproductive angiogenesis via STAT1 neddylation–DLL4/Notch axis. UBE2M-mediated neddylation promotes STAT1 phosphorylation and enhances its transcriptional activity, thereby upregulating DLL4 expression. In tip cells, where neddylation levels are elevated, increased DLL4 expression activates Notch signaling in adjacent stalk cells, suppressing their sprouting capacity. Endothelial deletion of UBE2M reduces DLL4\ expression, disrupts lateral inhibition, and leads to excessive nonproductive angiogenesis with impaired perfusion, ultimately inhibiting tumor growth.

The transmembrane ligand DLL4, expressed in tip cells, is a key Notch ligand that mediates lateral inhibition to control cell fate decisions and sprouting patterning. This study demonstrates that the upregulated neddylation pathway in tip cells regulates DLL4 via STAT1 modification to ensure tip-stalk cell homeostasis. Importantly, endothelial cell–specific deletion of UBE2M downregulates DLL4, driving excessive tip cell generation with inadequate stalk cell support. This imbalance stimulates sprouting but inhibits lumen extension, promoting nonproductive angiogenesis. The resultant vascular abnormalities intensify intratumoral hypoxia, triggering necrosis and apoptosis, resulting in suppressed tumor growth (Figure 10A).

Additionally, our study reveals that UBE2M mediates neddylation of STAT1 at K703, enhancing its transcriptional activity and promoting DLL4 expression. Intriguingly, while STAT1 was previously reported to undergo SUMOylation at the same residue in macrophages, a modification associated with reduced phosphorylation (58–60), we propose that STAT1 is differentially modified by neddylation versus SUMOylation at K703 in a cell type–specific manner or that these posttranslational modifications compete to regulate its activity. This pattern of lysine residue sharing between neddylation and SUMOylation is exemplified by the RPL11 protein, where their mutually antagonistic modification directly governs its nucleolar localization and function in stress signaling (61). Based on this established example, we propose that the upregulation of neddylation components in tumor endothelium thereby favors STAT1 neddylation, supporting tip cell sprouting through this modification-switching mechanism to execute functional programs that are precisely adapted to distinct cell types and microenvironmental demands. Future studies should validate this competitive model and elucidate how the dynamic balance between these modifications regulates STAT1 function across microenvironments.

Critically, we found that combining endothelial UBE2M inhibition with the VEGFR2 inhibitor (cediranib) produces a markedly stronger suppression of tumor growth in two mouse models, compared with individual treatments. This synergistic effect is consistent with findings in previous studies indicating that DLL4 expression may contribute to resistance against VEGF-targeted therapy (62), and DLL4/Notch blockade can inhibit the growth of anti-VEGF–resistant tumors and restore their treatment sensitivity. Supporting this, related studies have shown that dual targeting of DLL4 and VEGF in combination with chemotherapy further suppresses tumor growth (63–66). Further mechanistic investigation revealed that while endothelial UBE2M deletion alone resulted in the formation of dense but nonfunctional vasculature, the addition of cediranib reduced vessel density and partially enhanced pericyte coverage. This phenotypic shift likely represents a key mechanism behind the observed synergy. Additionally, UBE2M deletion in endothelial cells markedly elevated intratumoral hypoxia, which may induce VEGF expression and remodel the tumor microenvironment, thereby sensitizing tumors to subsequent anti-VEGF therapy. These insights position UBE2M inhibition as a promising strategy to enhance the efficacy of antiangiogenic therapies. Future efforts should focus on identifying predictive biomarkers, with endothelial neddylation levels, DLL4 expression, and STAT1 phosphorylation levels representing key candidates to identify patient selection for this combination approach.

In summary, our work elucidates what we believe to be a previously unrecognized role of the neddylation pathway, specifically through UBE2M-mediated modification of STAT1, in fine-tuning DLL4 expression and ensuring functional angiogenesis via tip-stalk cell homeostasis. Based on the clinical translational potential of neddylation inhibitors (such as the E1 inhibitor MLN4924, which has entered clinical trials) (67) (ClinicalTrials.gov NCT03965689), our study provides a targeting strategy for antitumor angiogenesis therapy and sensitization to anti-VEGF therapy.

## Methods

### Sex as a biological variable.

Our study examined male and female animals, and similar findings are reported for both sexes.

### Immunofluorescence image analysis.

For quantification of UBE2M expression in tumor endothelial cells, endothelial cells were identified by CD31 staining, and regions of interest were defined based on the CD31 signal using ImageJ (NIH) software. The mean fluorescence intensity of the UBE2M channel within these CD31-defined areas was then measured. The number of samples per experiment is indicated in the corresponding figure legends. At least 3 fields were analyzed per sample.

### Mice.

*Ube2m*^fl/fl^ mice were a gift from Yi Sun (Zhejiang University, Hangzhou, China) and have been described previously (68). *Ube2m*^fl/fl^ mice were crossed with Cdh5-CreERT2 mice to generate *Ube2m*^fl/fl^: Cdh5-CreERT2 (*Ube2m*^iECKO^, C57BL/6) mice. The *Ube2m*^fl/fl^: Cdh5-CreERT2 mice were i.p. injected with tamoxifen (20 mg/kg, i.p.) for 5 days, and effective knockout of UBE2M in endothelial cells was achieved after a 7-day interval. *Ube2m*^fl/fl^ mice injected with the same dose and duration of tamoxifen served as controls. Genotyping was performed by PCR of DNA samples using the following primers: *Ube2m*^fl/fl^ forward, CCGTGTCGTGAAGATTGTGAAGG; *Ube2m*^fl/fl^ reverse, ACCTCCACTGTCCTTCCTCGTCTC; Cdh5-CreERT2 forward, CCAAAATTTGCCTGCATTACCGGTCGATGC; Cdh5-CreERT2 reverse, ATCCAGGTTACGGATATAGT. All mice were kept under specific pathogen–free conditions. All animal protocols were approved by the Animal Care and Use Committee of the Zhejiang University School of Medicine.

### Cells.

HEK293T cells were purchased from the Chinese Academy of Sciences. Mouse B16 melanoma cells, LLC, and E0771 breast cancer cells were purchased from ATCC and cultured in DMEM basal medium (Gibco, Thermo Fisher Scientific) supplemented with 10% FBS and 1% penicillin/streptomycin. Non–small cell lung cancer (NSCLC) cells (A549, H1299) purchased from ATCC were grown in RPMI-1640 (Gibco, Thermo Fisher Scientific) with 10% FBS.

HUVECs were isolated from human umbilical veins (69, 70). After rinsing the umbilical cord with cold PBS, the vein was perfused with 0.2% (w/v) collagenase solution and digested at 37°C for 20 minutes. The cells were then collected and resuspended in complete M199 medium. HUVECs between passages 3 and 6 were used for experiments and cultured in complete M199 medium supplemented with 10% FBS, 1%P/S, 53% M199 (Gibco, Thermo Fisher Scientific), 37% human endothelial serum-free medium (Thermo Fisher Scientific), and 15 mg/mL endothelial cell growth supplement (Sigma-Aldrich).

The knockdown and overexpression of UBE2M in HUVECs were achieved through lentivirus infection. HEK293T cells were transfected with PSP (Addgene plasmid 12260), PMD (Addgene plasmid 12259), and PLVX-PURO (Takara Bio plasmid 632164) containing shRNA/UBE2M sequences (sequences are listed in Supplemental Table 3), and lentiviruses were harvested 48–72 hours later. The functional viral titer (TU/mL) was determined by flow cytometry–based quantification of ZsGreen^+^ HEK293T cells at 72 hours after transduction with serially diluted virus. The HUVECs were then infected with the lentivirus-containing supernatant and selected with puromycin to establish stable cell lines. The efficiency of UBE2M knockdown and overexpression was verified by qPCR and Western blotting.

### Mouse tumor models and treatment.

To achieve efficient UBE2M knockout, 6- to 8-week-old mice were i.p. injected with tamoxifen for 5 consecutive days, followed by a 1-week interval before tumor implantation. The subcutaneous syngeneic tumor model was established by injection of LLC or B16 (1×10^6^ cells in 100 μL PBS) cells subcutaneously into the dorsal flank of 8- to 10-week-old male mice. The orthotopic breast cancer model was established by inoculating E0771 cells (1 × 10^6^ cells in 100 μL PBS) into the mammary fat pads of 8-week-old female mice. Tumor growth became detectable at days 8–10 after implantation, after which tumor volumes were measured every other day. Tumors were harvested at approximately days 16–20 after implantation, weighed to determine tumor mass, and fixed in 4% paraformaldehyde (PFA). The tumor volume was calculated using the following formula: *V* = 0.5 × *L* × *W*², where *V* represents tumor volume (mm³), *L* denotes the longest diameter (mm), and *W* represents the perpendicular short diameter (mm). Cediranib (MCE) was administered daily via oral gavage at 1.5 mg/kg, starting after tumor growth was established and continuing until harvest.

To restore DLL4 expression in tumor endothelium, subcutaneous tumors were first established, followed by intratumoral injection of a Cre-inducible AAV-DLL4 upon observable growth. This was followed by 5 consecutive days of i.p. tamoxifen administration to induce UBE2M knockout and initiate DLL4 expression. Tumor volumes were measured every other day, and tumors were weighed at harvest.

### Western blotting, IP, and immunofluorescence staining.

For Western blotting, cells were lysed with RIPA lysis buffer (Beyotime Biotechnology) on ice, followed by incubation at 37°C for 30 minutes. The lysates were separated by SDS-PAGE and subsequently transferred to nitrocellulose membranes (Pall). Target proteins were finally detected using specified primary and secondary antibodies.

For IP of neddylated proteins, cells were lysed with 1% SDS, followed by DNA shearing via sonication and 5-minute denaturation at 100°C. The lysate was then diluted 10-fold with IP lysis buffer. Antibody-conjugated magnetic beads were incubated with the lysate overnight at 4°C, washed with PBST (0.05%Tween-20 in PBS) 3 times, and eluted with 1× SDS loading buffer for subsequent Western blot analysis.

For immunofluorescence of tissue samples, samples were fixed in 4% PFA, paraffin-embedded, and sectioned at 4 μm thickness. Following deparaffinization and rehydration, antigen retrieval was performed using pH 9.0 EDTA buffer. Sections were then permeabilized with 0.5% Triton X-100 for 15 minutes, blocked with 5% FBS for 1 hour at room temperature, and incubated with primary antibodies overnight at 4°C. After incubation with appropriate fluorescent secondary antibodies for 1 hour at room temperature, nuclei were stained with DAPI for 10 minutes, and samples were mounted with fluoromount-G (Southern Biotech) for fluorescence preservation. The images were further processed using ImageJ (NIH) 1.49v, FV31S-SW 2.1. or OlyVIA 3.1 (Olympus).

The following primary antibodies were used for Western blotting, IP, and immunofluorescence: anti-CD31 (Servicebio, GB150217-100; R&D Systems, AF3628); anti-UBE2M (Santa Cruz Biotechnology, sc-390064); anti-DLL4 (Cell Signaling Technology, 2589; R&D Systems, AF1389); anti-STAT1 (Cell Signaling Technology, 9172); anti-p-STAT1 (Santa Cruz Biotechnology, sc-8394); anti-CXCR4 (Abcam, ab124824); Hypoxyprobe-1 (Hypoxyprobe, Inc., HP1-100); and Ki67 (Abcam, ab15580).

### Quantitative PCR.

Total RNA was isolated from HUVECs using TRIzol reagent (ThermoFisher Scientific) and reverse-transcribed to cDNA using the ReverTraAce qPCR RT kit (Toyobo Inc.). Quantitative RT-PCR was performed using SYBR Green dye on a LightCycler 480 II Real-Time PCR System (Roche) with the primers listed in Supplemental Table 2. Gene expression was calculated using the 2^−ΔΔCt^ method and normalized to β-actin or GAPDH.

### Pimonidazole stain, vascular leakage and perfusion assay.

When tumor volume reached approximately 1,500 mm³, mice were intravenously injected with 100 μL Hypoxyprobe-1 (Hypoxyprobe) (60 mg/kg) to assess tumor hypoxia. After 60-minute circulation, mice were sacrificed and perfused with 1% PFA. Collected tumors were fixed, paraffin-embedded, sectioned, and stained with FITC-conjugated anti-pimonidazole monoclonal antibody for fluorescence detection.

To evaluate tumor vascular permeability and perfusion, mice were intravenously injected with 100 μL FITC-conjugated dextran (25 mg/mL, 70 kDa, Sigma-Aldrich), DyLight 488–conjugated Tomato lectin (1 mg/mL, Vector Laboratories), or DyLight 649–conjugated Tomato lectin (1 mg/mL, Thermo Fisher Scientific). Animals were perfused with 1% PFA to remove intravascular tracers after 30 minutes. Tumors were then harvested for further immunofluorescence analysis.

### Micro-CT angiography.

To reconstruct the 3D architecture of tumor vasculature, systemic vascular perfusion was performed with Microfil (Flow Tech). Following euthanasia, a thoracotomy was performed, and the systemic vasculature was perfused via the left ventricle with 20 mL PBS containing heparin, followed by 5 mL Microfil at a controlled infusion rate of 0.5 mL/min. Mice were incubated at 4°C overnight to allow polymer curing, after which tumors were harvested, fixed in 4% PFA, and subjected to micro-CT imaging for 3D vascular network reconstruction.

### Matrigel plug assay.

For the Matrigel angiogenesis assay, growth factor–reduced Matrigel (Corning) was mixed with recombinant mouse FGF-2 (400 ng/mL), VEGF-A (400 ng/mL), and heparin (50 units/mL). The mixture was subcutaneously injected into the right flank of mice. Seven days after implantation, the Matrigel plugs were harvested, photographed, and fixed in 4% PFA.

### Transwell cell migration assay.

Cultured HUVECs were trypsinized, resuspended in complete M199 medium, and seeded into the upper chamber of a Transwell (Corning). After 4-hour attachment, the upper chamber medium was replaced with serum-starved M199 (0.5% FBS) while the lower chamber received 600 μL complete medium. Following 24-hour incubation (37°C), nonmigrated cells in the upper chamber were removed, migrated cells were fixed with 4% PFA and stained with crystal violet (Beyotime). After imaging, stained cells were solubilized in 33% acetic acid for OD570 measurement.

### Wound healing migration assays.

HUVECs were seeded in 6-well plates (8×10^5^ cells/well) using complete M199 medium and cultured for 24 hours at 37°C. A uniform wound was created by scratching the monolayer with a 200 μL pipette tip, followed by PBS washing to remove detached cells. M199 medium with 0.5%FBS was added to each well, and cell migration was documented by microscopy at 4 and 6 hours after wounding.

### Tube formation assays.

For the angiogenesis assay, growth facto–-reduced Matrigel (Corning) was evenly coated in μ-Slide Angiogenesis plates (Ibidi) at 10 μL/well and polymerized at 37°C for 30 minutes. HUVECs were resuspended in M199 medium containing 0.5% FBS at 2×10^5^ cells/mL, and 50 μL cell suspension was carefully added to each Matrigel-coated well. After 3- to 5-hour incubation, tube formation was assessed by microscopy. For enhanced visualization, live cells were stained with calcein (Yeasen).

### Sprouting assays.

HUVECs were trypsinized, resuspended in complete M199 medium, and incubated with microcarrier beads (Cytiva, 37°C, 4 h) with intermittent resuspension every 20 minutes. After overnight culture, HUVEC-coated beads were washed and resuspended with fibrinogen (Solarbio) solution (2 mg/mL in PBS) containing 0.09 U/mL aprotinin (Sigma-Aldrich). Prepare fibrin gel by mixing 60 μL bead suspension with 40 μL thrombin (Sigma-Aldrich) solution (10 U/mL) in 96-well plates, followed by polymerization for 20 minutes. Sprouting was monitored for 20-48 hours after adding 100 μL complete M199 medium per well.

### Dual-luciferase reporter assay.

For the dual-luciferase reporter assay, a 2.1-kb human *DLL4* promoter fragment (obtained from GeneCopoeia) was cloned into the pGL3-Basic vector and cotransfected with STAT1 overexpression plasmid and pRL-TK control vector into HEK293T cells. After 24- to 48-hour incubation, firefly and Renilla luciferase activities were measured using the Dual-Luciferase Reporter Assay System (Promega).

### Human samples.

Tissue samples from 29 patients with lung cancer were obtained. The study protocol was approved by the Ethics Committee of Sir Run Run Shaw Hospital, Zhejiang University. All diagnoses were confirmed by specialized pathologists. The baseline characteristics of all participants are shown in Supplemental Table 1.

### Statistics.

Statistical analysis was performed using Prism software (GraphPad, version 9.0). All quantitative data are presented as the mean ± SEM. Unpaired 2-tailed Student’s *t* tests were used for comparisons between 2 groups. Multiple group comparisons were conducted by 1-way or 2-way ANOVA, followed by Tukey’s post hoc tests or multiple comparisons. *P* < 0.05 was considered statistically significant.

### Study approval.

All animal experiments were approved by the Animal Care and Use Committee of the Zhejiang University School of Medicine (ZJU20240934). The use of human tissue sections in the study was approved by the Ethical Committee of Sir Run Run Shaw Hospital (no. 2025-2661-01). This study was performed in compliance with all relevant ethical regulations, and all participants provided informed consent.

### Data availability.

Figure 1E was generated from the NCBI’s Gene Expression Omnibus (GEO) database GSE118904 (71). Figure 4O and Supplemental Figure 7, A–C were generated from the NCBI’s GEO database GSE159013 (72). The transcription factor prediction data were generated by JASPAR (73), UCSC Genome Browser (74), and TFDB (75). The remaining data supporting the findings of this study are available within the article, supplemental materials, or the Supporting Data Values file.

## Author contributions

YK, HC, Jie Zhang, and XJ conceived the study and designed the experiments. XJ and Jie Zhang performed most of the experiments, assisted by LZ, NS, and ZL. Jie Zhang, XX, Jisong Zhang, YH, EC, and XZ provided funding, technical, and material support. ZL performed bioinformatics analysis. XJ analyzed the data. XJ, YK, and HC wrote the manuscript. All authors reviewed the manuscript and provided final approval for submission.

## Conflict of interest

The authors have declared that no conflict of interest exists.

## Funding support

- National Natural Science Foundation of China (32570853 to HC, 32370920 to XZ, 82271862 to YK, and 32000799 to Jie Zhang).
- Zhejiang Province Natural Science Foundation of China (LZ23H020002 to HC, ZCLQN25H0101 to Jisong Zhang, and LZ26H160004 to Jie Zhang).

## Supplementary Material

Supplemental data

Supporting data values

## Version 1 07/21/2026

In-Press Preview

## Version 2 09/15/2026

Electronic publication
