# Supplementary material for: Endothelial deletion of the neddylation E2 enzyme UBE2M inhibits tumor growth by promoting nonproductive angiogenesis
Source: J Clin Invest. 2026 Jul 21;136(18):e201769. doi: 10.1172/JCI201769 (PMC13574159; doi:10.1172/JCI201769)
Supplement: Supplementary file 1 [file jci-136-201769-s257.pdf]

Supplemental Materials for

Endothelial deletion of the neddylation E2 enzyme UBE2M inhibits tumor growth by promoting non-productive angiogenesis

Xinyi Jiang[*1], Jie Zhang[*#2], Li Zhou[1], Zonglin Li[3], Ningcong Sun[1], Xian Xu[4], Jisong Zhang[4], Yizhou Huang[5], Xue Zhang[1,7], Enguo Chen[#4], Hongqiang Cheng[#3,6,7], Yuehai Ke[#1,7]

# Correspondence to: yke@zju.edu.cn; hqcheng11@zju.edu.cn; 3195024@zju.edu.cn and 3319019@zju.edu.cn.

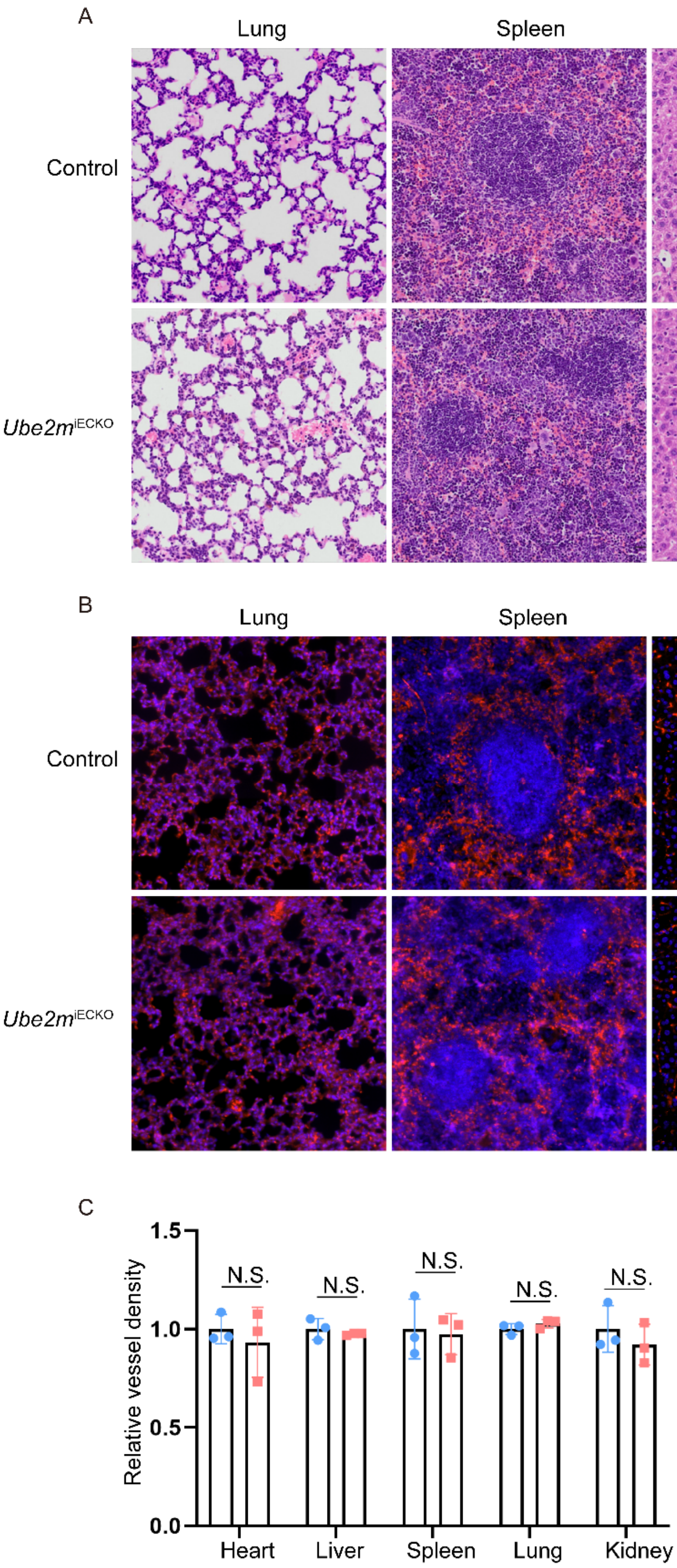
A
Lung
Spleen
Liver
Heart
Kidney
Control
Ube2m^iECKO
B
Lung
Spleen
Liver
Heart
Kidney
Control
Ube2m^iECKO
C
Relative vessel density
1.5
1.0
0.5
0.0
N.S.
N.S.
N.S.
N.S.
N.S.
Heart
Liver
Spleen
Lung
Kidney

Supplemental Figure 1. UBE2M deletion in adult mouse endothelia does not affect microvasculature in various organs. (**A**) H&E staining images showing histological morphology of lungs, spleens, livers, hearts and kidneys of *Ube2m*$^{fl/fl}$ and *Ube2m*^{iECKO} mice (n = 3). Scale bar: 100 μm. (**B**-**C**) Immunofluorescence images of $CD31^{+}$ vessels in lungs, spleens, livers, hearts and kidneys of *Ube2m*$^{fl/fl}$ and *Ube2m*^{iECKO} mice (n = 3). Scale bar: 50 μm. All mice received tamoxifen treatment as described in Methods.

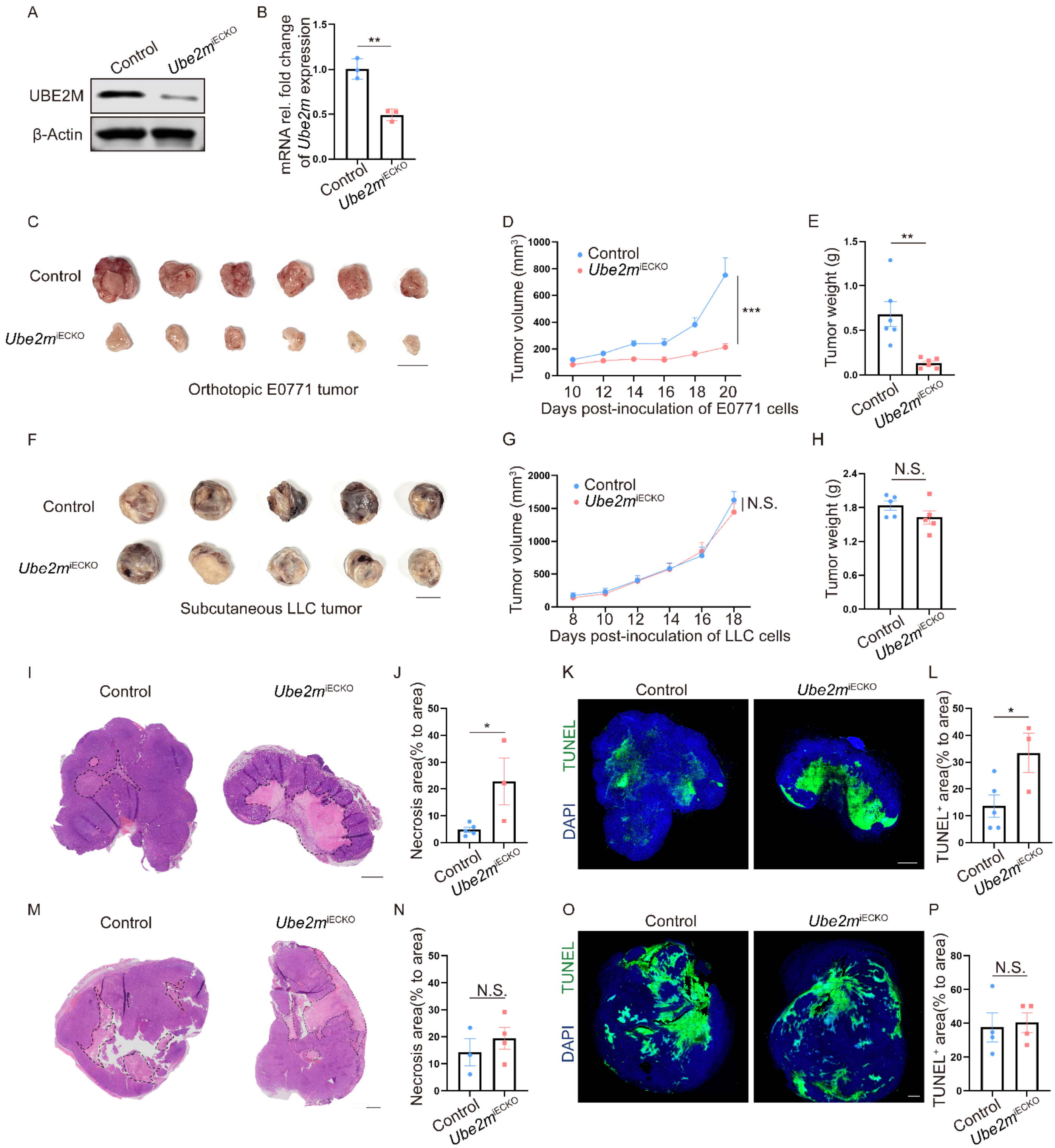
A
Control
Ube2m iECKO
UBE2M
β-Actin
B
mRNA rel. fold change of Ube2m expression
**
C
Control
Ube2m iECKO
Orthotopic E0771 tumor
D
Tumor volume (mm3)
Days post-inoculation of E0771 cells
***
E
Tumor weight (g)
**
F
Control
Ube2m iECKO
Subcutaneous LLC tumor
G
Tumor volume (mm3)
Days post-inoculation of LLC cells
N.S.
H
Tumor weight (g)
N.S.
I
J
Necrosis area(% to area)
*
K
TUNEL
DAPI
L
TUNEL+ area(% to area)
*
M
N
Necrosis area(% to area)
N.S.
O
TUNEL
DAPI
P
TUNEL+ area(% to area)
N.S.

16

Supplemental Figure 2. Endothelial UBE2M deletion suppresses orthotopic E0771 breast tumor growth but not subcutaneous LLC tumors. (**A**-**B**) Western blot (**A**) and qPCR (**B**) for UBE2M in lung endothelial cells from *Ube2m*$^{fl/fl}$ and *Ube2m*^{iECKO} mice. (**C**) Images for orthotopic E0771 tumors in *Ube2m*$^{fl/fl}$ and *Ube2m*^{iECKO} mice (n=6). Scale bar: 10 mm. (**D**-**E**) The volumes (**D**) and weights (**E**) of orthotopic E0771 tumors in *Ube2m*$^{fl/fl}$ and *Ube2m*^{iECKO} mice (n=6). (**F**) Images for subcutaneous LLC tumors in *Ube2m*$^{fl/fl}$ and *Ube2m*^{iECKO} mice (n=5). Scale bar: 10 mm. (**G**-**H**) The volumes (**G**) and weights (**H**) for LLC tumors in *Ube2m*$^{fl/fl}$ and *Ube2m*^{iECKO} (n=5). (**I**-**L**) Representative images of orthotopic E0771 tumor necrosis (**I**-**J**) and apoptosis (**K**-**L**). H&E staining was conducted and the necrosis area was labeled with dotted lines. Scale bar: 500 μm. Apoptosis was measured by TUNEL staining. Scale bar: 500 μm. (**M**-**P**) Representative images of LLC tumor necrosis (**M**-**N**) and apoptosis (**O**-**P**). H&E staining was conducted and the necrosis area was labeled with dotted lines. Scale bar: 1 mm. Apoptosis was measured by TUNEL staining. Scale bar: 1 mm. Data were shown as mean ± SEM. $*P < 0.05$; $**P < 0.01$; $***P < 0.001$; nonsignificant (N.S.), $P > 0.05$. Two-tailed Student's t-test (**B**, **E**, **H**, **I**-**P**); two-way ANOVA followed by Tukey's post hoc test (**D**, **G**).

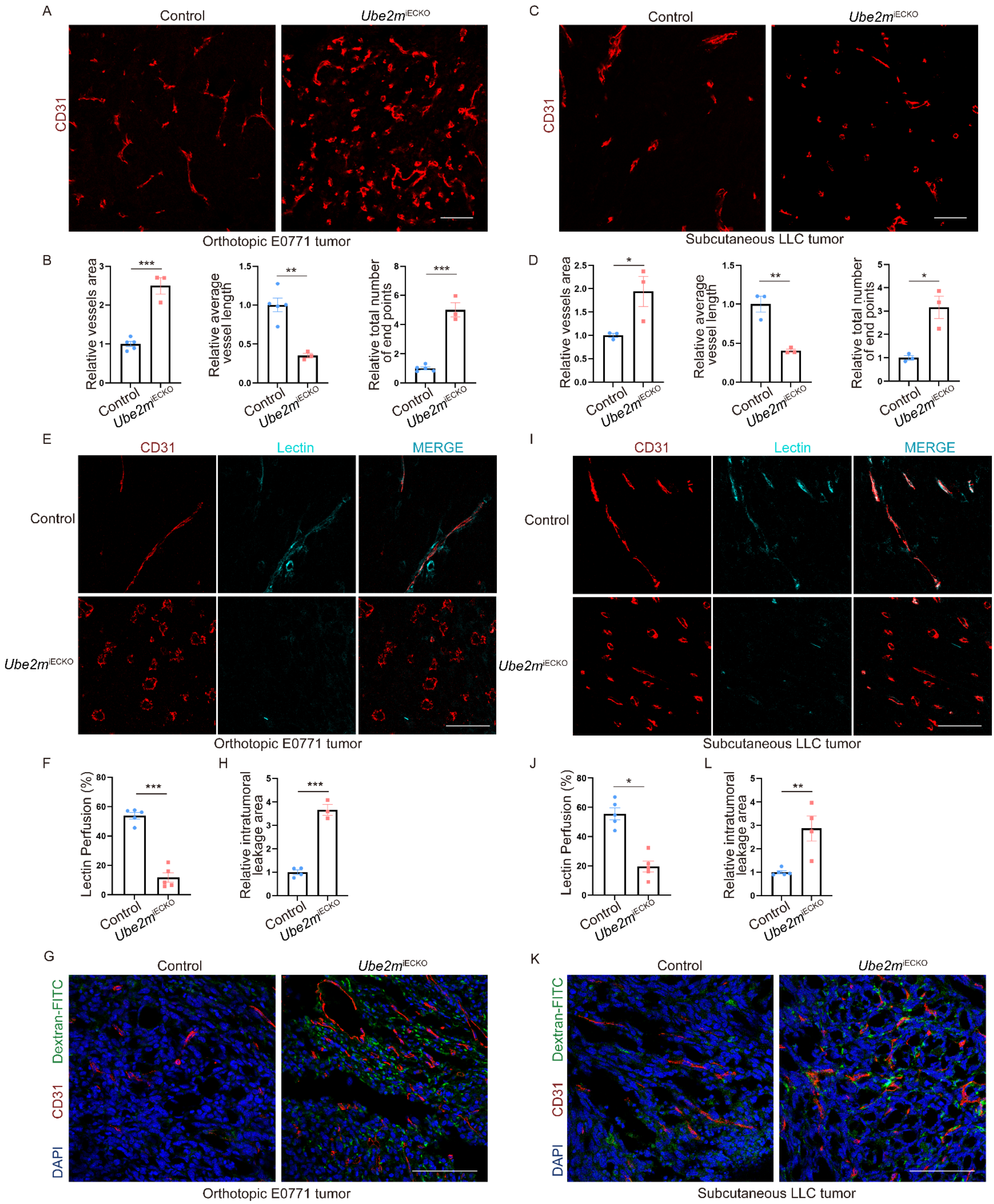

A
Control
Ube2m iECKO
CD31
Orthotopic E0771 tumor
C
Control
Ube2m iECKO
CD31
Subcutaneous LLC tumor
B
Relative vessels area
Relative average vessel length
Relative total number of end points
Control
Ube2m iECKO
D
Relative vessels area
Relative average vessel length
Relative total number of end points
E
CD31
Lectin
MERGE
Control
Ube2m iECKO
Orthotopic E0771 tumor
I
CD31
Lectin
MERGE
Control
Ube2m iECKO
Subcutaneous LLC tumor
F
Lectin Perfusion (%)
H
Relative intratumoral leakage area
J
Lectin Perfusion (%)
L
Relative intratumoral leakage area
G
Control
Ube2m iECKO
Dextran-FITC
CD31
DAPI
Orthotopic E0771 tumor
K
Control
Ube2m iECKO
Dextran-FITC
CD31
DAPI
Subcutaneous LLC tumor

Supplemental Figure 3. UBE2M deletion in endothelial cells promotes non-productive angiogenesis. (**A**-**B**) Immunofluorescence images of CD31 in orthotopic E0771 tumors in *Ube2m*$^{fl/fl}$ (n = 5) and *Ube2m*^{iECKO} (n = 3) mice. Images were analyzed by the Image J software. Scale bar: 50 μm. (**C**-**D**) Immunofluorescence images of CD31 in subcutaneous LLC tumor from *Ube2m*$^{fl/fl}$ (n = 3) and *Ube2m*^{iECKO} (n = 3) mice. Images were analyzed by the Image J software. Scale bar: 50 μm. (**E**-**F**) Immunofluorescence images of CD31 and Lectin in orthotopic E0771 tumors (n = 5). Lectin perfusion was measured by using Image J software. Scale bar: 100 μm. (**G**-**H**) Representative images for vessels perfused with dextran in orthotopic E0771 tumors from *Ube2m*$^{fl/fl}$ (n = 4) and *Ube2m*^{iECKO} (n = 3) mice. Images were analyzed by the Image J software. Scale bar: 100 μm. (**I**-**J**) Immunofluorescence images of CD31 and Lectin in subcutaneous LLC tumors (n = 5). Lectin perfusion was measured by using Image J software. Scale bar: 100 μm. (**K**-**L**) Representative images for vessels perfused with dextran in subcutaneous LLC tumor from *Ube2m*$^{fl/fl}$ (n = 5) and *Ube2m*^{iECKO} (n = 4) mice. Images were analyzed by the Image J software. Scale bar: 100 μm. Data are shown as means ± SEM. *P < 0.05; **P < 0.01; ***P < 0.001. p-values were generated by using the two-tailed Student's t-test.

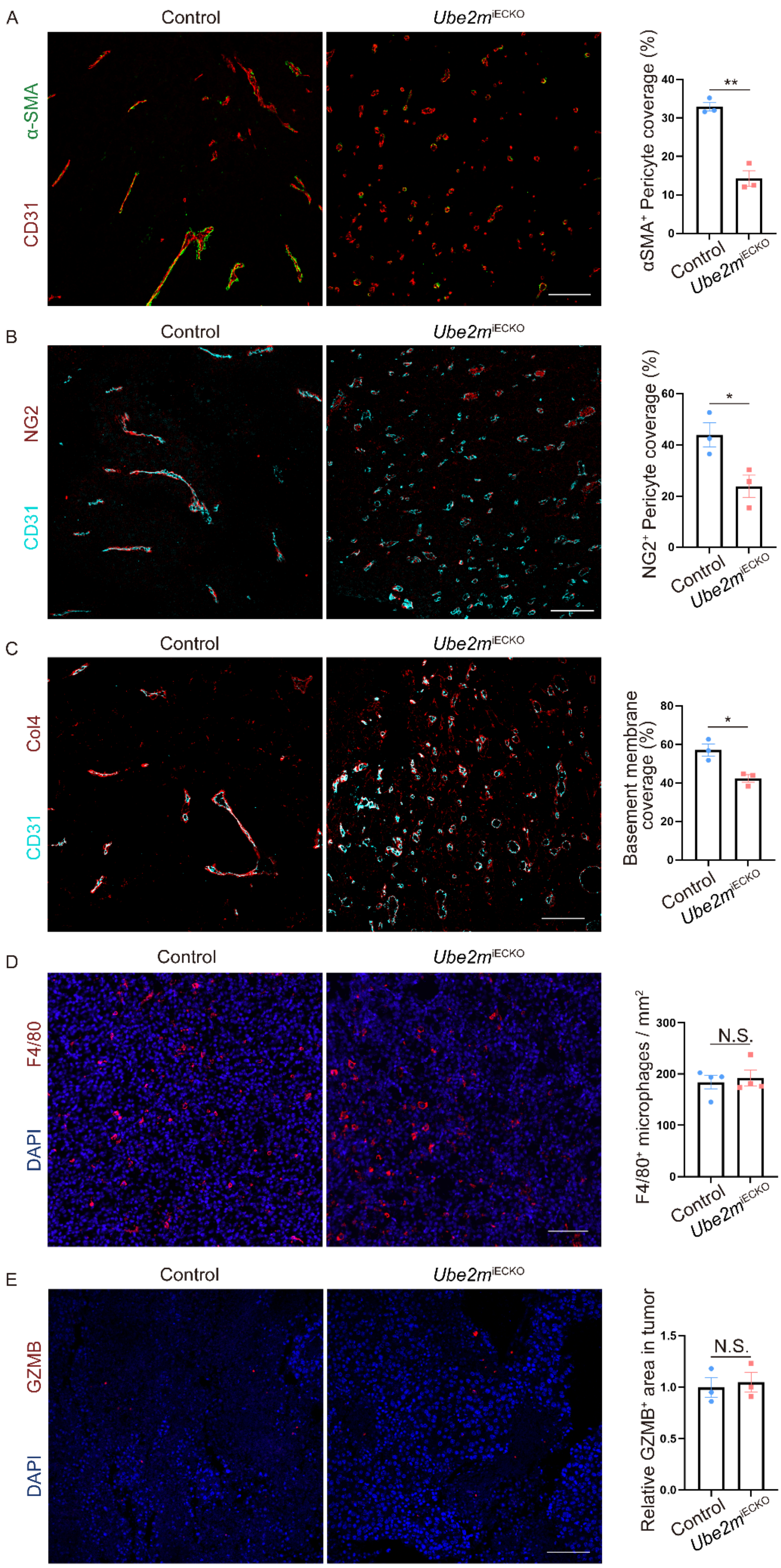

A
Control
Ube2m^iECKO
α-SMA
CD31
αSMA⁺ Pericyte coverage (%)
**
B
Control
Ube2m^iECKO
NG2
CD31
NG2⁺ Pericyte coverage (%)
*
C
Control
Ube2m^iECKO
Col4
CD31
Basement membrane coverage (%)
*
D
Control
Ube2m^iECKO
F4/80
DAPI
F4/80⁺ microphages / mm²
N.S.
E
Control
Ube2m^iECKO
GZMB
DAPI
Relative GZMB⁺ area in tumor
N.S.

Supplemental Figure 4. Endothelial UBE2M deficiency impairs pericyte and basement membrane coverage in tumor vasculature. (**A**-**C**) Immunofluorescence images of CD31, αSMA (**A**), NG2 (**B**), and collagen IV (**C**, Col4) in B16 tumors. Images were analyzed by the Image J software. n = 3 in each group. Scale bar: 100 μm. (**D**-**E**) Representative images for F4/80 (**D**, n = 4) and GZMB (**E**, n = 3) in B16 tumors. Images were analyzed by the Image J software. Scale bar: 100 μm. Quantitative data were shown as mean ± SEM. *P < 0.05; **P < 0.01; nonsignificant (N.S.), P > 0.05. p-values were generated by two-tailed Student's t-test.

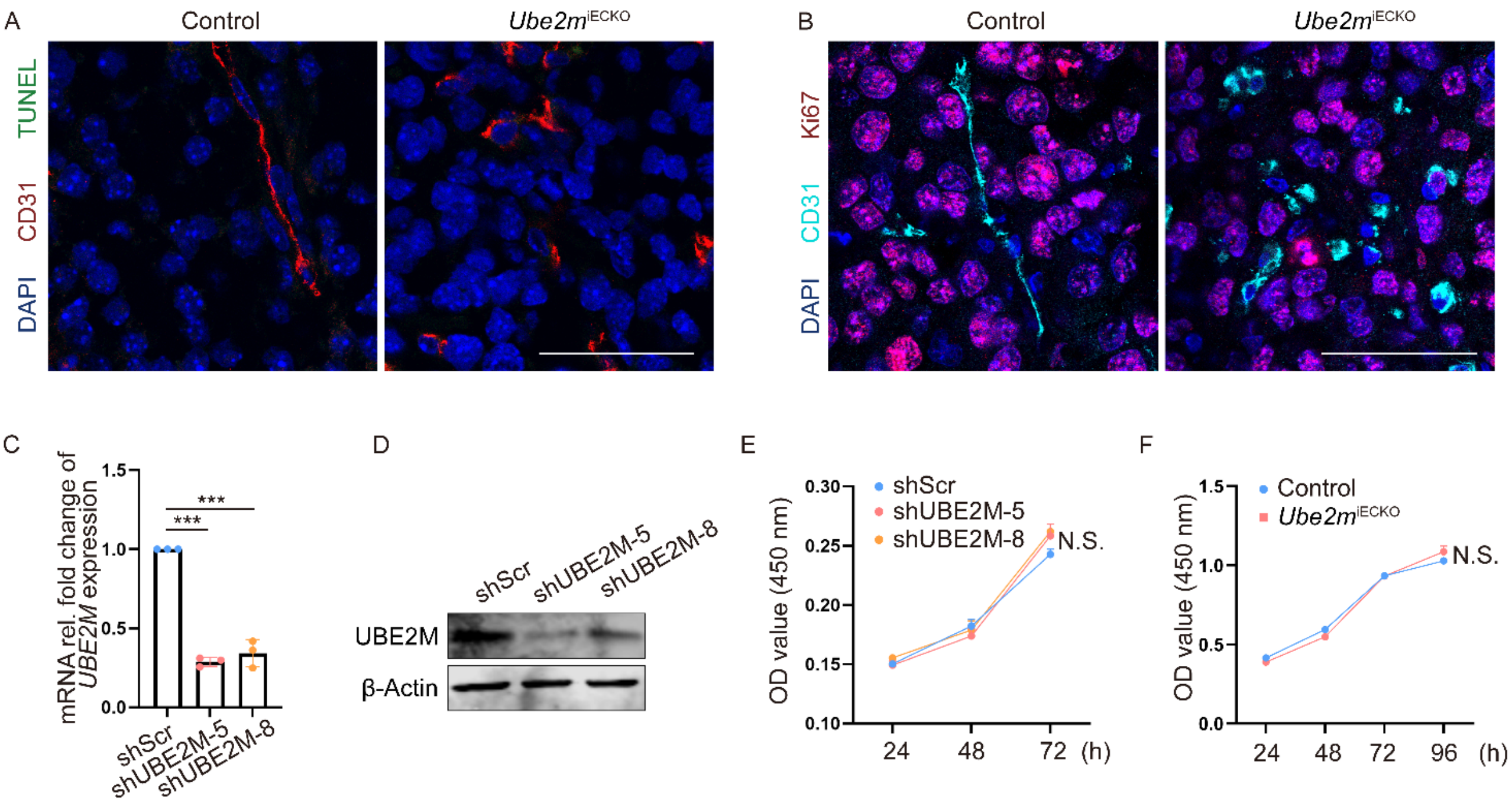

Supplemental Figure 5. UBE2M deficiency does not affect endothelial cell proliferation or apoptosis. (**A**) Representative images of TUNEL and CD31 co-staining in tumor sections from control and *Ube2m*^{iECKO} mice. Scale bar: 50 μm. (**B**) Representative images of Ki67 and CD31 co-staining in tumor sections from control and *Ube2m*^{iECKO} mice. Scale bar: 50 μm. (**C**-**D**) qPCR and western blot for UBE2M expression in HUVECs. (**E**-**F**) Cell proliferation of HUVECs with UBE2M knockdown (n = 3) and of mouse lung endothelial cells (MLECs) isolated from *Ube2m*$^{fl/fl}$ and *Ube2m*^{iECKO} mice (n = 3), was measured by CCK8 assay. two-way ANOVA followed by Tukey's post hoc test (**E**, **F**); one-way ANOVA followed by Tukey's post hoc test (**C**).

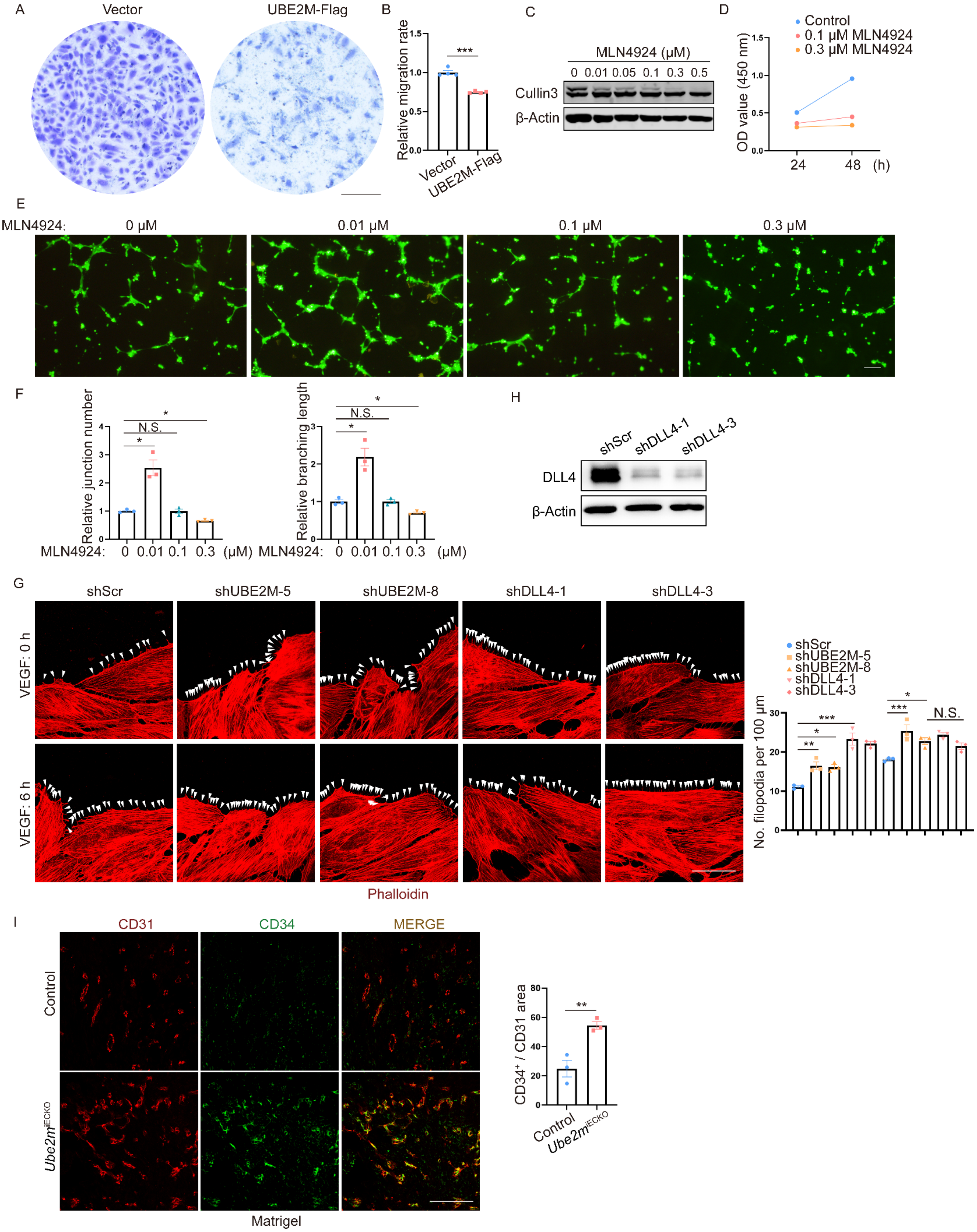
A
Vector
UBE2M-Flag
B
Relative migration rate
***
Vector
UBE2M-Flag
C
MLN4924 (μM)
0 0.01 0.05 0.1 0.3 0.5
Cullin3
β-Actin
D
Control
0.1 μM MLN4924
0.3 μM MLN4924
OD value (450 nm)
24 48 (h)
E
MLN4924:
0 μM
0.01 μM
0.1 μM
0.3 μM
F
Relative junction number
Relative branching length
N.S.
MLN4924: 0 0.01 0.1 0.3 (μM)
H
shScr
shDLL4-1
shDLL4-3
DLL4
β-Actin
G
shScr
shUBE2M-5
shUBE2M-8
shDLL4-1
shDLL4-3
VEGF: 0 h
VEGF: 6 h
Phalloidin
No. filopodia per 100 μm
I
CD31
CD34
MERGE
Control
Ube2m^ECKO
Matrigel
CD34⁺ / CD31 area
**

Supplemental Figure 6. Inhibition of neddylation leads to a tip cell phenotype in endothelial cells. (**A**-**B**) Cell migration measured by transwell migration (n = 4) assay in vitro of HUVECs. (**C**) Western blot for Cullin3 in MLN4924-treated HUVECs. (**D**) CCK8 assay of MLN4924-treated HUVECs. (**E**-**F**) Tube formation assay in HUVECs treated with a concentration gradient of MLN4924 (n = 3). Scale bar: 100 μm. (**G**) The images and comparison of filopodia formation in endothelial cells (n = 3). White arrows indicate phalloidin-positive filopodia. Scale bar: 50 μm. (**H**) Western blot for DLL4 expression in DLL4-knockdown and control HUVECs. (**I**) Representative images showing CD34 level in $CD31^+$ vessels in matrigel plugs (n = 3). Scale bar: 100 μm. The $CD34^+$ area in endothelial cells was analyzed by the Image J software. Data were shown as mean ± SEM. *P < 0.05; **P < 0.01; ***P < 0.001; nonsignificant (N.S.), P > 0.05. Two-tailed Student's t-test (**B**, **I**); two-way ANOVA followed by Tukey's post hoc test (**D**); one-way ANOVA followed by Tukey's post hoc test (**F**, **G**).

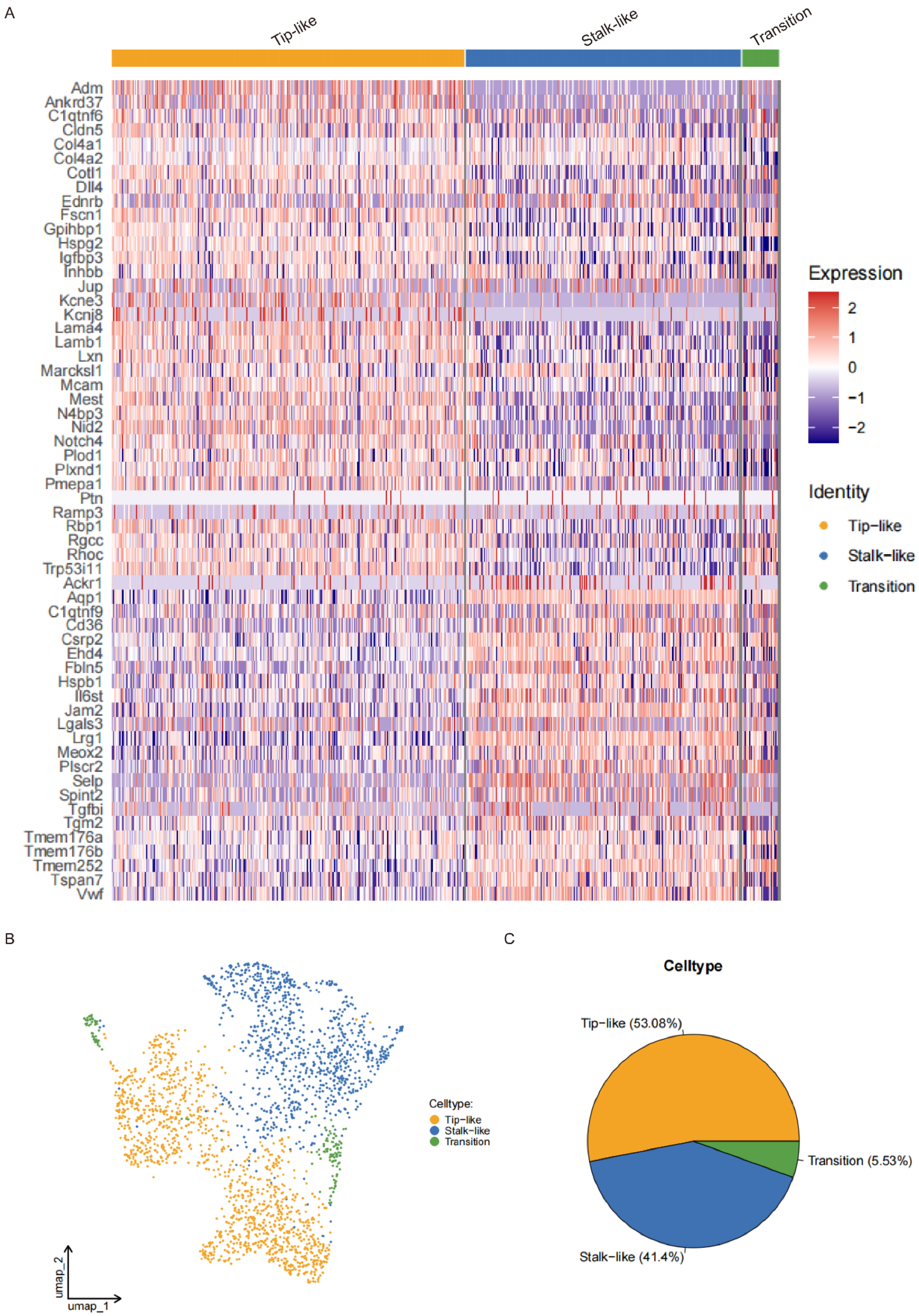
A
Tip-like
Stalk-like
Transition
Adm
Ankrd37
C1qtnf6
Cldn5
Col4a1
Col4a2
Cotl1
Dll4
Ednrb
Fscn1
Gpihbp1
Hspg2
Igfbp3
Inhbb
Jup
Kcne3
Kcnj8
Lama4
Lamb1
Lxn
Marcksl1
Mcam
Mest
N4bp3
Nid2
Notch4
Plod1
Plxnd1
Pmepa1
Ptn
Ramp3
Rbp1
Rgcc
Rhoc
Trp53i11
Ackr1
Aqp1
C1qtnf9
Cd36
Csrp2
Ehd4
Fbln5
Hspb1
Il6st
Jam2
Lgals3
Lrg1
Meox2
Plscr2
Selp
Spint2
Tgfbi
Tgm2
Tmem176a
Tmem176b
Tmem252
Tspan7
Vwf
Expression
2
1
0
−1
−2
Identity
Tip−like
Stalk−like
Transition
B
Celltype:
Tip−like
Stalk−like
Transition
umap_2
umap_1
C
Celltype
Tip−like (53.08%)
Transition (5.53%)
Stalk−like (41.4%)

Supplemental Figure 7. Characterization and classification of tumor endothelial cell (EC) subtypes. (**A**) Heatmap showing the expression of canonical marker genes used to classify ECs into three distinct subtypes: tip cells, stalk cells, and transitional cells. (**B**) UMAP projection visualizing the endothelial cell clusters, colored by subtype. (**C**) A pie chart quantifying the proportional abundance of each EC subtype within the dataset. Data source: GSE159013.

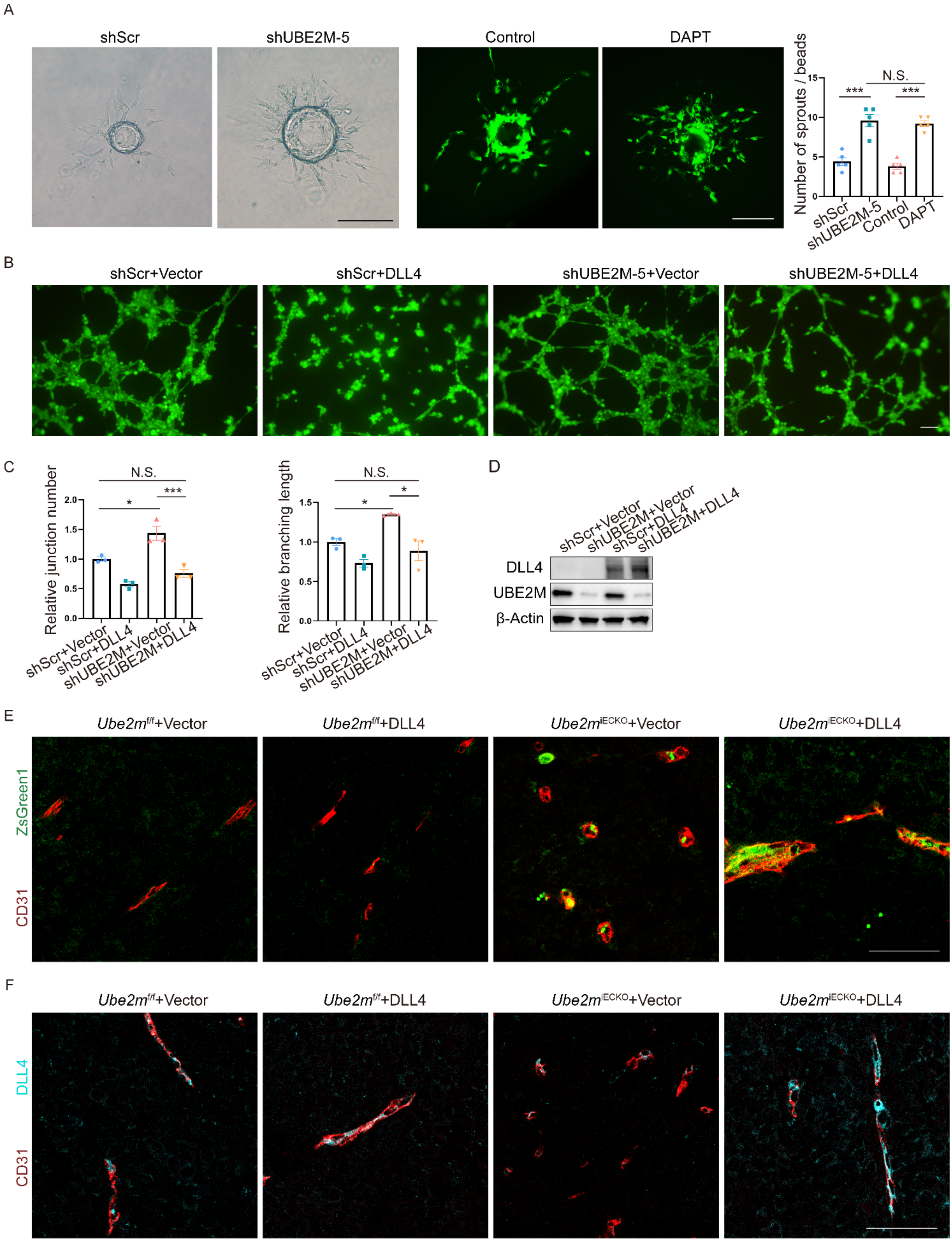
A
shScr
shUBE2M-5
Control
DAPT
Number of sprouts / beads
N.S.
***
***
shScr
shUBE2M-5
Control
DAPT
B
shScr+Vector
shScr+DLL4
shUBE2M-5+Vector
shUBE2M-5+DLL4
C
Relative junction number
N.S.
*
***
shScr+Vector
shScr+DLL4
shUBE2M+Vector
shUBE2M+DLL4
Relative branching length
N.S.
*
*
shScr+Vector
shScr+DLL4
shUBE2M+Vector
shUBE2M+DLL4
D
shScr+Vector
shUBE2M+Vector
shScr+DLL4
shUBE2M+DLL4
DLL4
UBE2M
β-Actin
E
Ube2m f/f+Vector
Ube2m f/f+DLL4
Ube2m iECKO+Vector
Ube2m iECKO+DLL4
ZsGreen1
CD31
F
Ube2m f/f+Vector
Ube2m f/f+DLL4
Ube2m iECKO+Vector
Ube2m iECKO+DLL4
DLL4
CD31

Supplemental Figure 8. Reconstitution of DLL4 in UBE2M-knockdown endothelial cells results in phenotypic rescue. (**A**) Sprouting assay of UBE2M-knockdown and DAPT-treated endothelial cells compared with normal endothelial cells. (**B-C**) Tube formation assay in HUVECs after UBE2M knockdown and subsequent DLL4 rescue. (**D**) Western blot for DLL4 in UBE2M-knockdown HUVECs with DLL4 re-expression. (**E**) Representative images for GFP in B16 tumors in *Ube2m*^{iECKO} mice. GFP was not shown in *Ube2m*$^{fl/fl}$ mice without Cre recombinase. Scale bar: 100 μm. (**F**) Representative immunofluorescence images of CD31 and DLL4 in B16 tumors. Scale bar: 50 μm.*$P < 0.05$; ***$P < 0.001$; nonsignificant (N.S.), $P > 0.05$. One-way ANOVA followed by Tukey’s post hoc test (**C**).

A

MS results of HUVEC with
Nedd8-Flag overexpression

Protein	Peptides(#)	Coverage(%)
TRIM21	22	42
Cullin3	19	29
UBA3	16	46
Cullin2	15	20
Cullin4A	15	22
Cullin1	13	19
Cullin4B	13	14
NAE1	11	25
UBE2M	4	23
SMURF2	3	5
Beta-catenin	2	3
STAT1	3	6

B

MS results of HUVEC with
Nedd8L73P-Flag overexpression

Protein	Peptides(#)	Coverage(%)
TRIM21	25	48
Cullin2	32	37
Cullin3	26	38
Cullin1	20	25
Cullin4A	16	26
Cullin4B	14	16
UBA3	10	28
UBE2M	8	37
Cullin5	7	8
NAE1	7	17
UBE2F	3	19
SMURF2	3	3
Beta-catenin	3	4
STAT1	3	5
STAT3	1	3

C

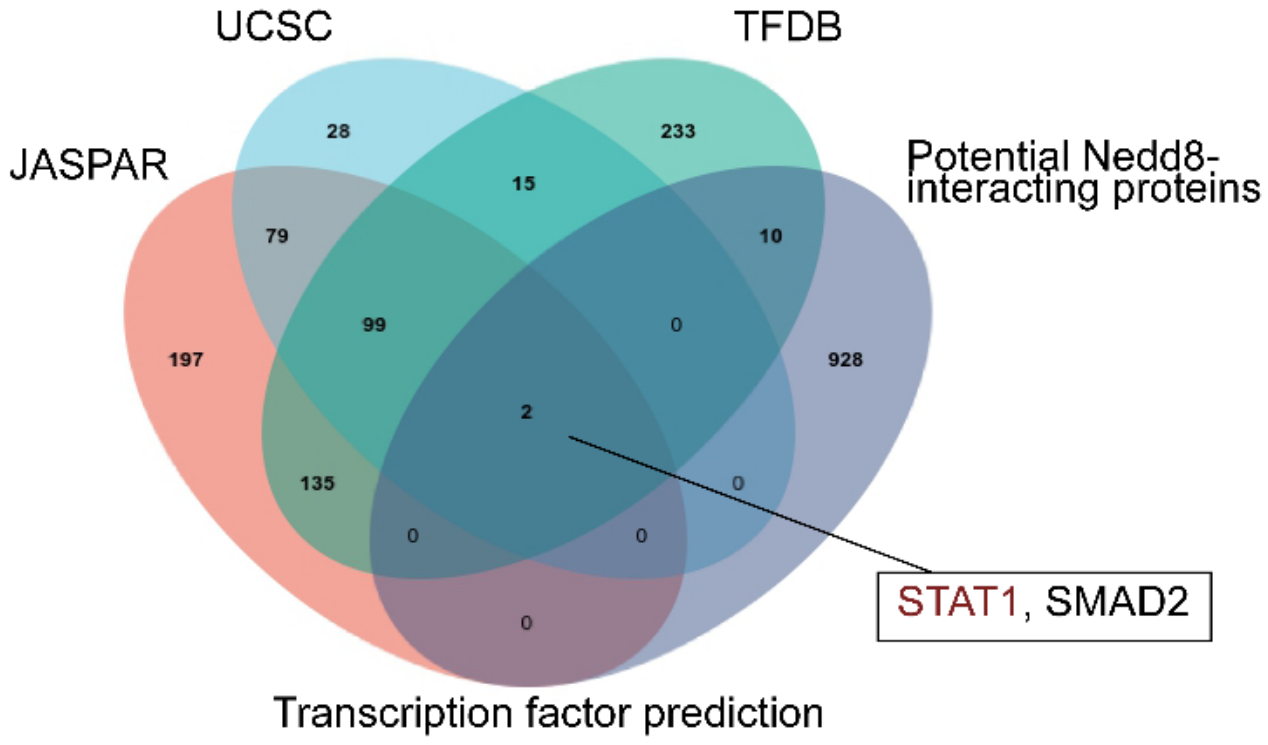

D

# NeddyPreddy (v0.3) – Prediction Results

Identifier	Residue	Class
sp|P42224|STAT1_HUMAN	110	Neddylated
sp|P42224|STAT1_HUMAN	673	Neddylated
sp|P42224|STAT1_HUMAN	679	Neddylated
sp|P42224|STAT1_HUMAN	697	Neddylated
sp|P42224|STAT1_HUMAN	703	Neddylated

E

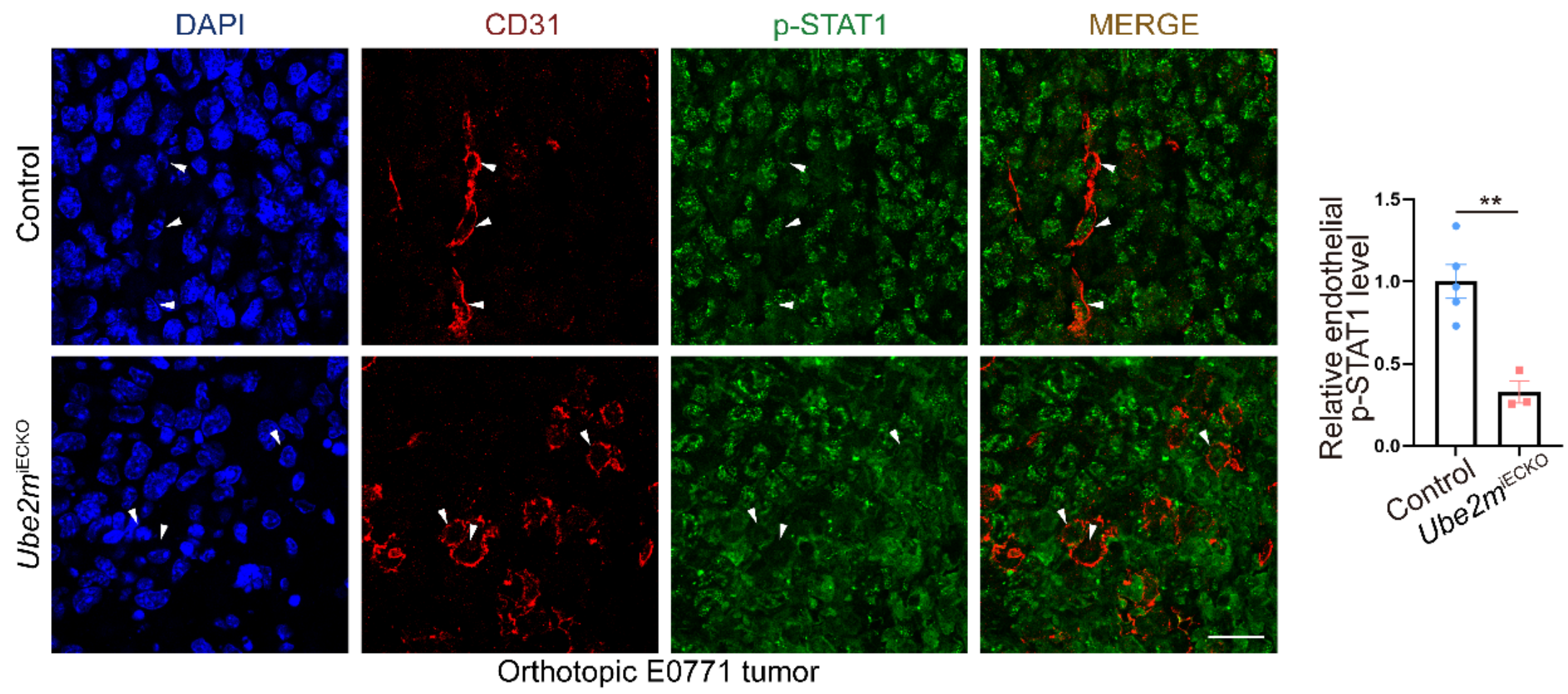

F

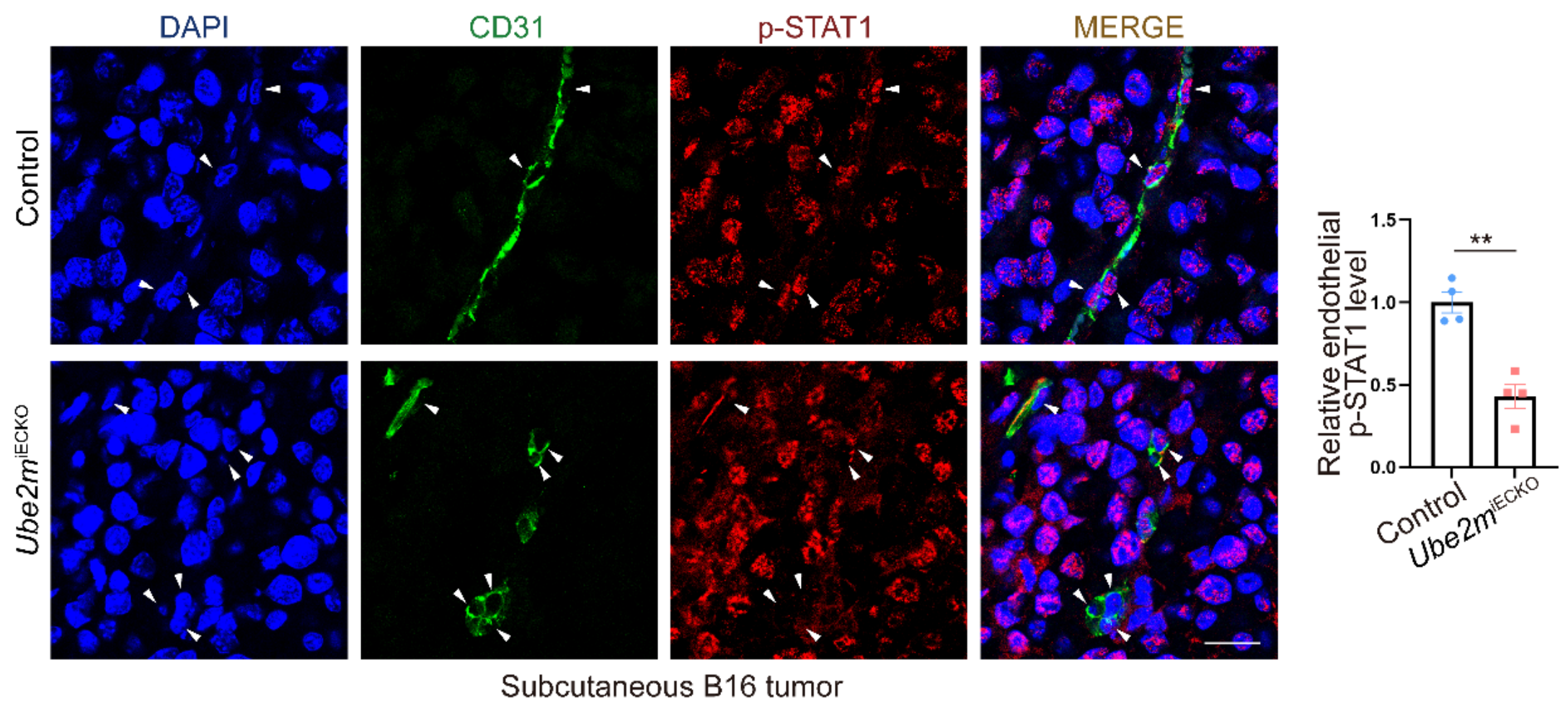

Supplemental Figure 9. STAT1 is a potential substrate for neddylation

(**A**-**B**) Representative mass spectrometry results of potential NEDD8-conjugated proteins in HUVECs**.** (**C**) Overlap of predicted DLL4 transcription factors (TFs) from three databases with the NEDD8 interactome in HUVECs identified by mass spectrometry (MS). (**D**) Potential neddylated lysine sites of STAT1 predicted by NeddyPreddy. (**E**) Representative images showing p-STAT1 level in $CD31^+$ vessels of orthotopic E0771 tumors from *Ube2m*$^{fl/fl}$ and *Ube2m*^{iECKO} mice. n = 5 *Ube2m*$^{fl/fl}$ and n = 3 *Ube2m*^{iECKO} mice. Scale bar: 20 μm. Images were analyzed by Image J software. (**F**) Representative images showing p-STAT1 level in $CD31^+$ vessels of subcutaneous B16 tumors from *Ube2m*$^{fl/fl}$ and *Ube2m*^{iECKO} mice. n = 4 per group. Scale bar: 20 μm. Images were analyzed by Image J software. Quantitative data were shown as mean ± SEM. **P < 0.01. Two-tailed Student's t-test (**E-F**).

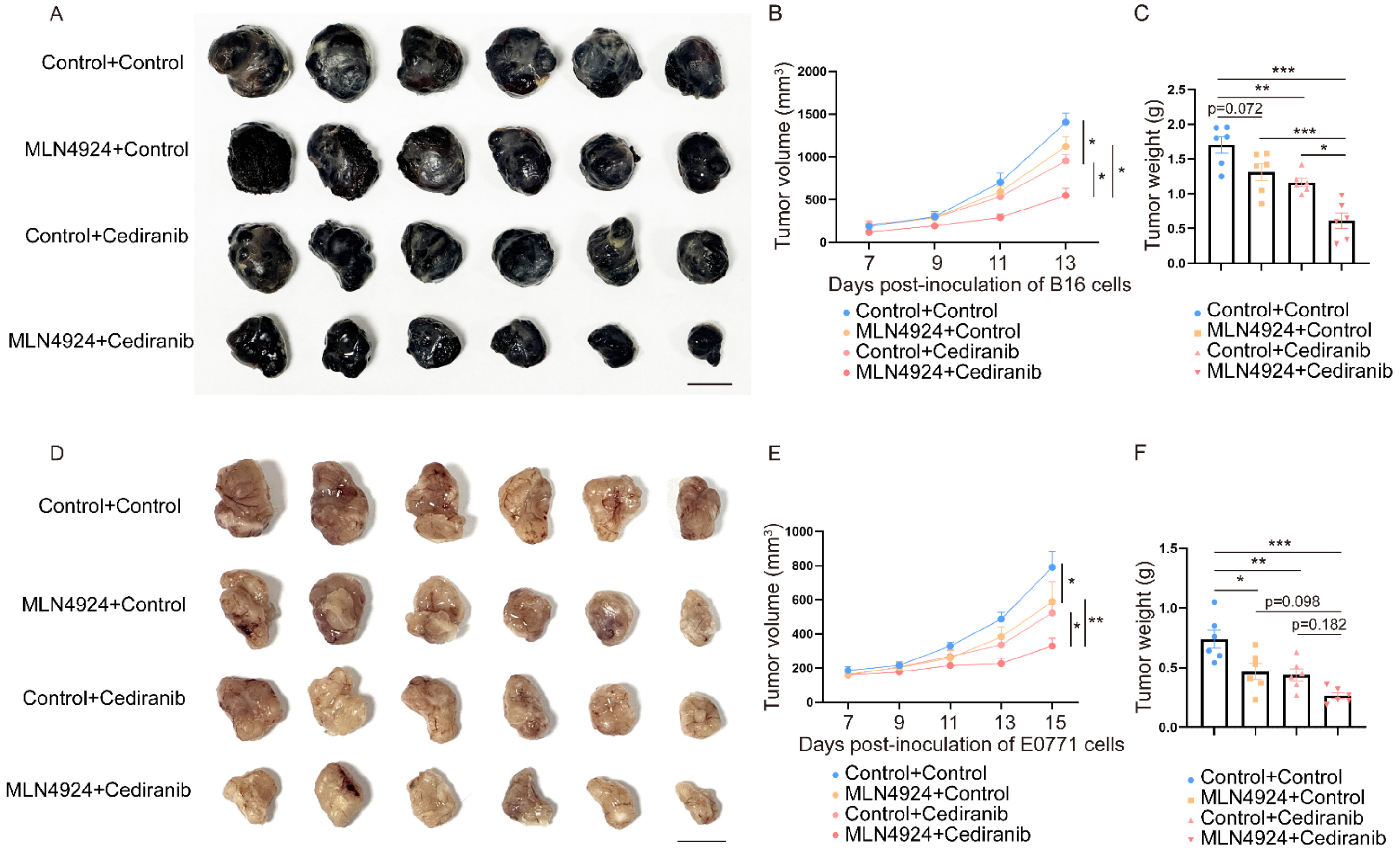

Supplemental Figure 10. Combined treatment with MLN4924 and Cediranib enhances anti-tumor efficacy. (**A**) Images of subcutaneous B16 tumors from mice treated with vehicle, MLN4924, Cediranib, or the combination of both drugs. Scale bar: 10 mm. (**B-C**) The volumes (**B**) and weights (**C**) of B16 tumors from treated with vehicle, MLN4924, Cediranib, or the combination of both drugs. n=6 per group. (**D**) Images of orthotopic E0771 tumors from mice treated with vehicle, MLN4924, Cediranib, or the combination of both drugs. Scale bar: 10 mm. (**E-F**) The volumes (**E**) and weights (**F**) of orthotopic E0771 tumors from mice treated with vehicle, MLN4924, Cediranib, or the combination of both drugs. n=6 per group. Data

were shown as mean ± SEM. *P < 0.05; **P < 0.01; ***P < 0.001. Two-way ANOVA with Tukey’s post hoc test (**B**, **E**) or one-way ANOVA followed by Tukey’s post hoc test (**C**, **F**).

Supplemental Table 1

Patient ID	Age	Sex	Pathological diagnosis
PT-01	58	Male	Pulmonary nodule
PT-02	61	Male	Pulmonary nodule
PT-03	52	Male	Pulmonary nodule
PT-04	27	Female	Pulmonary nodule
PT-05	52	Female	Pulmonary nodule
PT-06	69	Male	Lung adenocarcinoma
PT-07	73	Male	Lung adenocarcinoma
PT-08	79	Female	Lung adenocarcinoma
PT-09	77	Female	Lung adenocarcinoma
PT-10	63	Female	Lung adenocarcinoma
PT-11	82	Male	Lung adenocarcinoma
PT-12	76	Male	Lung adenocarcinoma
PT-13	72	Male	Lung adenocarcinoma
PT-14	50	Female	Poorly differentiated lung carcinoma
PT-15	65	Male	Poorly differentiated lung carcinoma
PT-16	72	Male	Poorly differentiated lung carcinoma
PT-17	86	Male	Poorly differentiated lung carcinoma
PT-18	79	Male	Poorly differentiated lung carcinoma
PT-19	31	Male	Poorly differentiated lung carcinoma

PT-20	33	Female	Poorly differentiated lung carcinoma
PT-21	76	Male	Poorly differentiated lung carcinoma
PT-22	77	Male	Small cell lung carcinoma
PT-23	65	Female	Small cell lung carcinoma
PT-24	69	Male	Small cell lung carcinoma
PT-25	73	Male	Small cell lung carcinoma
PT-26	71	Female	Small cell lung carcinoma
PT-27	79	Male	Small cell lung carcinoma
PT-28	66	Male	Small cell lung carcinoma
PT-29	68	Male	Small cell lung carcinoma

Supplemental Table 2

Primer Name	Primer sequence 5'-3'
Human-GAPDH-F	GTCAAGGCTGAGAACGGGAA
Human-GAPDH-R	GATGGCATGGACTGTGGTCA
Human-β-Actin-F	CAGGATGCAGAAGGAGATCACTG
Human-β-Actin-R	TACTCCTGCTTGCTGATCCACAT
Human-UBE2M-F	ATGAGGGCTTCTACAAGAGTGG
Human-UBE2M-R	ATTGTCTCACACTTCACCTTGG
Human-DLL4-F	GCCCTTCAATTTCACCTGGC
Human-DLL4-R	CAATAACCAGTTCTGACCCACAG
Human-STAT1-F	CGGCTGAATTTCGGCACCT
Human-STAT1-R	CAGTAACGATGAGAGGACCCT
Human-ANGTP1-F	CAGAAAGCTGACAGATGTTGAGACC
Human-ANGTP1-R	ACTCTTCCTTGTGTTTTCCTTCCA
Human-HES1-F	GCTACCCCAGCCAGTGTCAA
Human-HES1-R	TCTTGCTCTTCGTCTTTTCTCCA
Human-PDGFB-F	CTCGATCCGCTCCTTTGATGA
Human-PDGFB-R	CGTTGGTGCGGTCTATGAG
Human-SOX7-F	TCGACGCCCTGGATCAACT
Human-SOX7-R	CTGGGAGACCGGAACATGC
Human-NOTCH1-F	CGACAACGCCTACCTCTGCT

Human-NOTCH1-R	ACAGGCACACTCGTAGCCATC
Human-ERG-F	CCAGCGTCCTCAGTTAGATCCTT
Human-ERG-R	CATCTTGAACTCCCCGTTGGT
Human-TGFB1-F	CCTGGCGATACCTCAGCAAC
Human-TGFB1-R	GCTAAGGCGAAAGCCCTCAA
Human-ATX-F	AGAGCAGAAGGATGGGAGGAAG
Human-ATX-R	TCACAGCGACAATCAGGAGGT
Human-VEGFR3-F	AGAGACTTTGAGCAGCCATTCATC
Human-VEGFR3-R	GTCATCCCACACCACCTCCT
Human-FOXC2-F	GAGTCCCAGGTGAGTGGCAAT
Human-FOXC2-R	ATTTCGTGCAGTCGTAGGAGTAGG
Human-CTNNB1-F	CTTGGACTTGATATTGGTGCCCA
Human-CTNNB1-R	GGCCACCCATCTCATGTTCCATC
Human-TEAD2-F	CCGAAGGAAATCAAGGGAAA
Human-TEAD2-R	GAGATGAGCTGGGCAGAGGA
Human-YAP1-F	CAGGTTGGGAGATGGCAAAG
Human-YAP1-R	GGGCTGTGACGTTCATCTGG
Human-VEGFR2-F	CGCAGAGTGAGGAAGGAGGA
Human-VEGFR2-R	GGATGATGACAAGAAGTAGCCAGAA
Human-NR2F2-F	AGTGGGCATGAGACGGGAAG
Human-NR2F2-R	GACAGGTACGAGTGGCAGTTGAG

Human-TEK-F	ACCTCTTCACCTCGGCCTTC
Human-TEK-R	TCACACGTCCTTCCCATAAACC
Human-JAG1-F	CAGATTTCCTTGTTCCCTTGCT
Human-JAG1-R	CGTTGTTGGTGGTGTTGTCCT
Human-VEGFR1-F	TTTGCCTGAAATGGTGAGTAAGG
Human-VEGFR1-R	TGGTTTGCTTGAGCTGTGTTC
Human-FOXC1-F	GGAGATGTTCGAGTCACAGAGGA
Human-FOXC1-R	GACGTGCGGTACAGAGACTGG
Human-CYR61-F	TGCCGCCTTGTGAAAGAAAC
Human-CYR61-R	CTCAAACATCCAGCGTAAGTAAACC
Human-HEY1-F	AGGTTCCATGTCCCCAACTACA
Human-HEY1-R	TGCAGGATCTCGGCTTTTTC
Human-CTGF-F	TTCCAAGACCTGTGGGATGG
Human-CTGF-R	GGGAGTACGGATGCACTTTTTG
Human-NOTCH4-F	TCTCGTCCTCCAGCTCATCC
Human-NOTCH4-R	CATCACAACTCCATCCTCATCAAC
Human-FGFR2-F	AGCACCATACTGGACCAACAC
Human-FGFR2-R	GGCAGCGAAACTTGACAGTG
Human-VEGFA-F	GATGAGCTTCCTACAGCACAACAA
Human-VEGFA-R	TTTCGTTTTTGCCCCTTTCC
Human-DLL1-F	GAGCGTGGGGAGAAAGTGTG

Human-DLL1-R	ACTTGCATTCCCCTGGTTTGT
Human-HEY2-F	CGTCGGGATCGGATAAATAACA
Human-HEY2-R	CAAGAGCGTGTGCGTCAAAG
Human-TEAD4-F	CCAGTATGAGAGCCCCGAGA
Human-TEAD4-R	CGGTGGATGCGGTAAGAGTAG
Human-NRP1-F	GGAAACACCAACCCCACAGA
Human-NRP1-R	CATACCCAACATTCCAGAGCAAG
Human-TEAD1-F	GATGCTGGGGCTTTTTATGGT
Human-TEAD1-R	ACATTGGGGAGCGGTTTATTC
Human-EFNB2-F	CAAGTCCCTTTGTAAAACCAAATCC
Human-EFNB2-R	GGCGAGTGCTTCCTGTGTCT
Human-RBPJK-F	TACGAGTGTGGTTTGGGGATG
Human-RBPJK-R	GTAGGTAAAGGTAAGGCTGGTGGAA
Human-MEF2C-F	CAGGACAAGGAATGGGAGGA
Human-MEF2C-R	ACTGACTGAGGGCAGATGGTG
Human-TAZ-F	GTATCCCAGCCAAATCTCGTGATG
Human-TAZ-R	CAGCGCATTGGGCATACTCATG
Human-ETV2-F	CGATGCCCCAAAACTAACCA
Human-ETV2-R	TAATTCATGCCCGGCTTTCTC
Human-TEAD3-F	CCCCTAATGCCTTCTTCCTTGT
Human-TEAD3-R	TCTCCACCTTCTCTACCACCTGTTT

Human-LPP3-F	CTGCTCATCTGCCTCGACCT
Human-LPP3-R	CACAGCGTCATTTATTGTCTCACC
Human-PROX1-F	GGCTCTCCTTGTCGCTCATAAA
Human-PROX1-R	GGAGCTGGGATAACGGGTATAAAAA
Human-ANGPT2-F	GAACCAAACAGCGGAGCAAA
Human-ANGPT2-R	TCGAGAGGGAGTGTTCCAAGAG

Supplemental Table 3

shRNA	Sequence (5'-3')
shScr	CCTAAGGTTAAGTCGCCCTCG
shUBE2M-5	GGTCCCACCAGGCTATTAAAG
shUBE2M-8	GGTGAAGTGTGAGACAATGGT
shSTAT1-3	CTGGAAGATTTACAAGATGAA
shSTAT1-4	CGACAGTATGATGAACACAGT
shDLL4-1	GCATCTGCCTTAAGCACTTCC
shDLL4-3	GCCTTCCTTCTGCATTGTTTA
